# Engineering Heterogeneous Tumor Models for Biomedical Applications

**DOI:** 10.1002/advs.202304160

**Published:** 2023-11-09

**Authors:** Zhuhao Wu, Danqing Huang, Jinglin Wang, Yuanjin Zhao, Weijian Sun, Xian Shen

**Affiliations:** ^1^ Department of Rheumatology and Immunology Nanjing Drum Tower Hospital School of Biological Science and Medical Engineering Southeast University Nanjing 210096 China; ^2^ Department of Gastrointestinal Surgery The First Affiliated Hospital Wenzhou Medical University Wenzhou 325035 China; ^3^ Department of Gastrointestinal Surgery The Second Affiliated Hospital and Yuying Children's Hospital of Wenzhou Medical University Wenzhou 325027 China

**Keywords:** drug screening, microfluidics, organ‐on‐a‐chip, tissue engineering, tumor heterogeneity, tumor models

## Abstract

Tumor tissue engineering holds great promise for replicating the physiological and behavioral characteristics of tumors in vitro. Advances in this field have led to new opportunities for studying the tumor microenvironment and exploring potential anti‐cancer therapeutics. However, the main obstacle to the widespread adoption of tumor models is the poor understanding and insufficient reconstruction of tumor heterogeneity. In this review, the current progress of engineering heterogeneous tumor models is discussed. First, the major components of tumor heterogeneity are summarized, which encompasses various signaling pathways, cell proliferations, and spatial configurations. Then, contemporary approaches are elucidated in tumor engineering that are guided by fundamental principles of tumor biology, and the potential of a bottom‐up approach in tumor engineering is highlighted. Additionally, the characterization approaches and biomedical applications of tumor models are discussed, emphasizing the significant role of engineered tumor models in scientific research and clinical trials. Lastly, the challenges of heterogeneous tumor models in promoting oncology research and tumor therapy are described and key directions for future research are provided.

## Graphical abstract

This review focuses on the underlying fundamentals and applications of heterogeneous tumor engineering. It concludes the causes of tumor heterogeneity, including signal pathways, cell proliferation, and structures. It also discusses the potentials and advances of bottom‐up complex tumor engineering. Finally, it provides insights into the biomedical applications of heterogeneous tumor models, highlighting current challenges and outlining future directions.

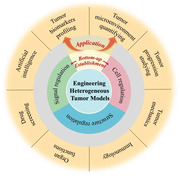

## 1 Introduction

Tumor development often leads to heterogeneities that can cause difficulties in oncology research and clinical treatments.^[^ ^1^, ^2^, ^3^, ^4^ ^]^ In recent decades, attention has been paid to tumor heterogeneity to facilitate the development of mechanism studying and therapy discovering.^[^ ^5^, ^6^, ^7^ ^]^ From an analytical perspective, the study of tumor heterogeneity can focus on its causes and consequences.^[^ ^8^, ^9^, ^10^ ^]^ For example, intratumoral complexity can cause heterogeneity among tumor cells, resulting from genomic instability,^[^ ^11^, ^12^, ^13^, ^14^, ^15^, ^16^ ^]^ abnormal transcriptome,^[^ ^17^, ^18^ ^]^ diverse cell populations,^[^ ^19^, ^20^, ^21^ ^]^ and altered extracellular matrix (ECM).^[^ ^22^, ^23^ ^]^ Specifically, unstable gene sequences^[^ ^24^, ^25^ ^]^ and transcriptome^[^ ^26^ ^]^ can activate specific signal pathways, while altered expression of cadherin on tumor cell membranes would significantly influence tumorigenesis and tumor progression.^[^ ^27^ ^]^ In addition, dynamic cellular components and altered ECM characteristics play an essential role in tumor proliferation, differentiation, invasion, and metabolism.^[^ ^28^, ^29^, ^30^, ^31^ ^]^ Consequently, the spatial structures, phenotypes, and functions of tumor tissues become highly heterogeneous, making tumors more complex and difficult to evaluate.^[^ ^32^, ^33^, ^34^ ^]^ Thus, a comprehensive understanding of the causes and consequences of tumor heterogeneity is critical for developing effective therapeutic strategies.^[^ ^35^, ^36^, ^37^, ^38^ ^]^

**Figure 1:**
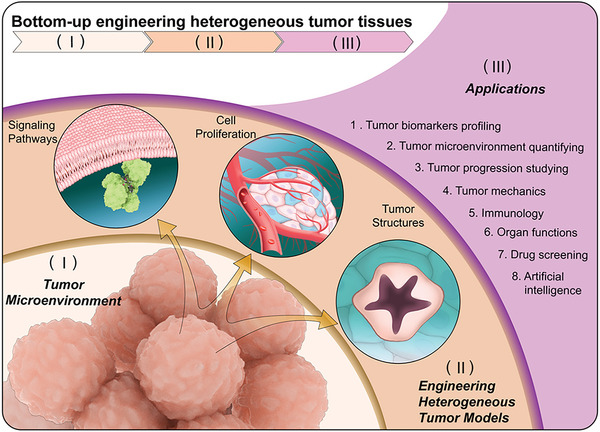
Schematic illustration of engineered heterogeneous tumor models for biomedical applications.

**Figure 2:**
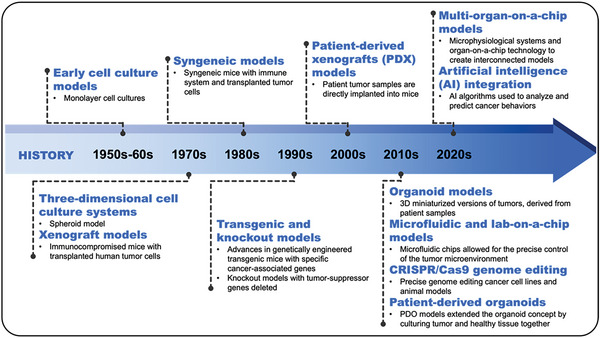
The history of tumor engineering. Since the establishment of the first human cancer cell line, the development of tumor models has experienced many important milestones.

To accurately assess tumor heterogeneity, it is imperative to engineer and characterize reliable tumor models.^[^ ^39^, ^40^, ^41^, ^42^ ^]^ Heterogeneous tumor engineering has gained significant momentum, primarily due to the need to study the fundamental relationship between tumor development and function.^[^ ^43^, ^44^, ^45^, ^46^ ^]^ Generally, engineering approaches rely on basic mechanisms to trigger the renewal potential of tumor tissues and study their morphodynamics (**Figure** **1**).^[^ ^47^, ^48^, ^49^ ^]^ In recent years, bioengineering techniques such as bioprinting^[^ ^50^, ^51^, ^52^, ^53^ ^]^ and micro‐electromechanical systems (MEMS)^[^ ^54^, ^55^, ^56^, ^57^, ^58^ ^]^ have been integrated into bottom‐up heterogeneous tumor engineering to create higher‐order developmental events, including long‐range tumor cell patterning, cellular and cell‐extracellular matrix (ECM) communications, and tumor‐tissue crosstalk.^[^ ^59^, ^60^, ^61^, ^62^ ^]^ Engineered heterogeneous tumor models have been exploited by various techniques, such as biosensors and physiological monitoring, to characterize the molecular, developmental, and mechanical properties of tumor tissues (**Figure** **2**).^[^ ^63^, ^64^, ^65^, ^66^ ^]^ Therefore, the establishment of bottom‐up tumor tissue models and real‐time evaluation of their properties would become the mainstream approach in tumor engineering, facilitating precision medicine and drug discovery.^[^ ^67^, ^68^ ^]^

This review provides an overview of the field of heterogeneous tumor engineering, focusing on the fundamental principles and regenerative potentials that underlie the development of high‐fidelity tumor models at multiple scales. From a fundamental perspective, we analyze the causes of tumor heterogeneity, including diverse signal pathways, cell proliferation, and structures. Then, we discuss different bottom‐up tumor bioengineering tools based on tumor developmental principles and growth potentials. In addition, we extend the overview of heterogeneous tumor engineering to include characterization techniques that provide valuable physiological information during tumor development. Moreover, we provide insights into the applications of heterogeneous tumor models, emphasizing the importance of efficient tumor interrogation in vitro. Finally, this review provides a comprehensive overview of the present obstacles and forthcoming pathways in heterogeneous tumor engineering.

## 2 Heterogeneity of Tumor Tissues

In order to advance the engineering of more effective tumor models, it is imperative to direct attention toward the heterogeneous nature of tumor tissues.^[^ ^69^, ^70^ ^]^ Tumor heterogeneity, which encompasses the stochastic distribution of genetically related signaling cues and cell populations, as well as the dynamic structures of extracellular matrix and framework in disease sites, contributes to the formation of specific tumor cell plasticity.^[^ ^2^, ^71^, ^72^, ^73^ ^]^ Cancer is characterized by the uncontrolled growth of various cells through oncogenic signaling pathways, with several significant signaling pathways implicated in the heterogeneity of cancer tissues during oncogenesis, structure, and progression.^[^ ^74^ ^]^ The roles of signaling pathways associated with VEGF, cadherin, and CAFs are particularly noteworthy in this context.^[^ ^75^, ^76^, ^77^ ^]^

In clinical settings, various subpopulations of intra‐tumor cells demonstrate distinct responses to drug treatments, ultimately leading to limited therapeutic efficacy.^[^ ^78^ ^]^ Consequently, a single cytotoxic therapy cannot effectively target all malignant cells. The pronounced and personalized heterogeneity of tumor tissues amongst patients highlights the crucial importance of precise evaluation of tumor heterogeneity in improving treatment efficacy.^[^ ^79^, ^80^ ^]^ Therefore, a fundamental understanding of tumor heterogeneity is indispensable for the in vitro reconstruction of multicellular and multidimensional tumor tissues, which may ultimately enhance treatment responses.

### 2.1 Signaling Pathways

**Figure 3:**
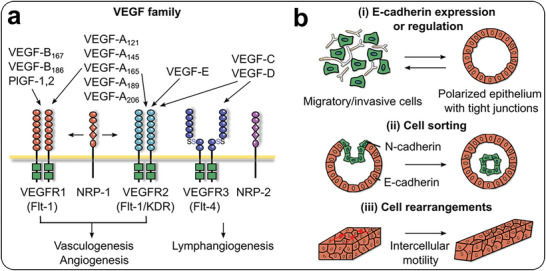
Critical signaling pathways for maintaining tumor homeostasis and heterogeneity. a) VEGF family and their receptors that influence the vasculogenesis, angiogenesis, and lymphangiogenesis in tumor tissues. b) Cadherin regulation in tissue morphogenesis including cell self‐organization, cell sorting, and rearrangement for tumor tissues.

Tumor growth is heavily reliant on the establishment of a neovasculature, which is essential for the delivery of oxygen and nutrients.^[^ ^81^ ^]^ The angiogenic process in tumors is a complex phenomenon, regulated by a network of pro‐ and anti‐angiogenic signals that intricately govern this process. Among the significant signaling pathways, the vascular endothelial growth factor (VEGF) family, encompassing proteins and receptors, assumes a pivotal role. This family comprises a group of growth factors, such as VEGF, placental growth factor (PlGF)−1, and PlGF‐2 for angiogenesis (**Figure** **3**a).^[^ ^82^ ^]^ VEGF‐A, a homodimeric glycoprotein with a mass of 45 kDa, encompasses five isoforms of 121, 145, 165, 189, and 206, possessing the capability to bind with high affinity to VEGFR1 and VEGFR2, respectively. VEGF‐A165, as the dominant isoform, is frequently overexpressed in various solid tumors, ultimately leading to vasculogenesis and angiogenesis. Furthermore, VEGF‐C and ‐D can bind onto VEGFR3 and mediate lymphangiogenesis. The absence of PlGF hampers angiogenesis, plasma extravasation, inflammation, and tumor growth.^[^ ^83^ ^]^ A more profound understanding of the VEGF‐VEGFR family can help develop novel therapeutic strategies, such as anti‐VEGF therapies, for various cancers.^[^ ^84^ ^]^

In the tumorigenesis and morphology of tumor tissues, signaling pathways play a crucial role, including the cadherin family of transmembrane glycoproteins.^[^ ^85^ ^]^ Cadherins regulate cell growth, cell‐cell adhesion, and the maintenance of homeostatic tissue structures.^[^ ^86^ ^]^ They dynamically regulate cell organization, cell sorting, and cell rearrangements (Figure 3b).^[^ ^87^ ^]^ Cadherins are important in the orderly turnover of fast renewal tissues like the stabilization of tissue architectures to prevent the dissociation of tumor cells and the organization of gut and epidermis.^[^ ^88^ ^]^ Specifically, epithelial cadherin (E‐cadherin) mediates cell adhesions in epithelial‐mesenchymal transition (EMT), which turns cells from a loose mesenchymal network into a polarized epithelial barrier comprising a tight junction. Loss or dysregulation of E‐cadherin can induce tumor cell invasion and metastasis. Furthermore, cadherins also regulate specific cell recognition processes and the formation of selective cell connections. Cadherin affinity shapes tissue morphology, with N‐cadherin and E‐cadherin having high homotypic affinity but low heterotypic affinity, resulting in the aggregation of cells with the same N‐ or P‐cadherin expression.^[^ ^89^ ^]^ Although cadherins are commonly regarded as mediating cell‐cell interactions and recognitions, they also regulate cell rearrangements, such as C‐cadherin being needed for the convergence and extension of tissue motions, contributing to the elongation of the body axis in gastrulating *X. laevis* embryos.^[^ ^90^ ^]^ Together, cadherins underlie tumor microenvironments, such as the specific distribution and stabilization of tumor cells.

### 2.2 Cell Proliferation

**Figure 4:**
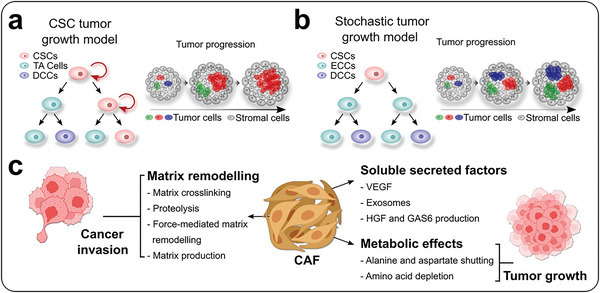
The role of cell proliferation in tumor progression. a) CSCs are the major proliferative cells during tumor progression. TA cells and DCCs show limited and nonproliferative potentials, respectively. Tumor tissues eventually exhibit homogeneous morphology. b) In the stochastic tumor growth model, all the cancer cells exhibit similar clonal expansion capacity. Tumor tissues eventually exhibit synergetically multidirectional proliferation. Embryonal carcinoma cells (ECCs). c) The role of CAFs to regulate tumor invasion and growth.

The tumor microenvironment comprises a complex ecosystem consisting of diverse cells, including proliferating cells, infiltrating inflammatory cells, CAFs, and so on.^[^ ^91^ ^]^ This section specifically emphasizes the pivotal contribution of proliferating cancer stem cells, called CSCs, in tumor development.^[^ ^92^, ^93^, ^94^, ^95^ ^]^ CSCs have been identified based on the histological heterogeneity observed in tumor tissues, as they can influence all cells and establish spatial structures in tumors (**Figure** **4**a).^[^ ^96^, ^97^ ^]^ Tumor growth is significantly influenced by the condition of CSCs, resulting in hierarchical and stochastic tumor growth models. In the first one, transit‐amplifying (TA) cells and dormancy‐competent CSCs (DCCs) have limited and nonproliferative potentials, respectively, whereas only CSCs exhibit strong self‐renewal ability and eventually dominate tumor progression.^[^ ^98^ ^]^ In the tumor stochastic model, cancer cells go through self‐renewal or differentiation into non‐proliferative counterparts, leading to similar expansion capacity (Figure 4b).^[^ ^99^ ^]^ Therefore, a better understanding of CSCs could facilitate the engineering of tumor tissues such as tumor organoids with high heterogeneity and plasticity, which could promote the investigation of the role of different cell types in tumor development.

Tumor microenvironment is a dynamic and multifaceted system that contains diverse cell types, including infiltrating inflammatory cells and cancer‐associated fibroblasts (CAFs), which are integral to regulating tumor homeostasis.^[^ ^100^ ^]^ Immunocytes are essential in tumor immune evasion. Their interaction with tumor cells can modulate tumorigenesis and reveal new therapeutic opportunities.^[^ ^101^ ^]^ Conversely, CAFs promote cancer invasion and metastasis by secreting matrix‐crosslinking enzymes that alter the extracellular matrix (ECM) to increase tissue stiffness.^[^ ^102^, ^103^ ^]^ Furthermore, CAFs secrete various signaling molecules, like TGF‐β, HGF, GAS6, and VEGF, which enhance tumor invasion, proliferation, and angiogenesis (Figure 4c). CAFs communicate with cancer cells by exchanging metabolites and amino acids, which can significantly impact immunoregulation and ultimately promote tumor growth.^[^ ^104^ ^]^ Therefore, tissue engineering approaches that can modulate cell proliferation and induce tumor heterogeneity from the bottom‐up may provide a valuable platform to decipher the distinct roles of different cell types in the tumor microenvironment.

### 2.3 Structures

**Figure 5:**
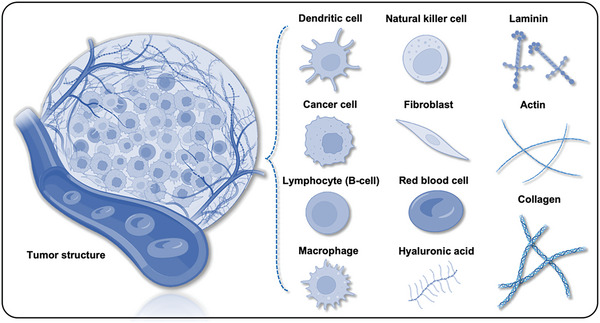
Structures of tumor tissues containing two distinct compartments: the parenchyma and the stroma.

In the realm of oncology, tumor growth is defined as the aberrant proliferation of cells, which gives rise to a mass or lump.^[^ ^105^, ^106^, ^107^, ^108^ ^]^ In this review, we concentrate on solid tumors, which exhibit an apparent structure that is similar to that of normal tissues.^[^ ^109^, ^110^ ^]^ Specifically, solid tumors are composed of two interconnected compartments: the parenchyma that consists of the neoplastic cells, and the stroma is that induced by the neoplastic cells and serves as their milieu (**Figure** **5**).^[^ ^111^ ^]^ The rapid proliferation of neoplastic cells and their consequent upregulation of pro‐angiogenic factors result in the formation of atypical vasculatures, which significantly differ from those observed in healthy tissues.^[^ ^112^ ^]^ In solid tumors, the stroma comprises connective tissue, blood vessels, and frequently inflammatory cells, which act as an intermediary between the malignant cells and the normal host tissues. The stroma is primarily a product of the host and is triggered as a consequence of the communications between tumor cells and the host.^[^ ^113^ ^]^ Vasculature is one of the primary constituents of the tumor stroma and is typically composed of a labyrinth of disorganized vessels that lack the conventional hierarchy of blood vessels, making it challenging to identify arterioles, capillaries, and venules.^[^ ^114^, ^115^ ^]^ The overall structure of tumors has genetic, epigenetic, and phenotypic effects on cancer cells, which guide and direct cancer progression.

## 3 Current Techniques for Bottom‐up Engineering of Heterogeneous Tumor Models

In the field of tumor engineering, the development of heterogeneous tumor models is a crucial step for advancing the understanding of tumor diseases.^[^ ^116^ ^]^ However, creating such models is challenging due to the absence of reliable manufacturing techniques capable of reconstructing the complex tumor microenvironment.^[^ ^117^, ^118^ ^]^ This microenvironment comprises a range of intrinsic gene mutations, diverse signal expressions, unique cell distributions, and spatial tissue structures, which collectively confer self‐renewal and self‐organization capabilities to the tumor tissue.^[^ ^45^ ^]^ These characteristics allow the growth of new tumors from even a small metastatic tumor fragment in distant sites. Consequently, bottom‐up strategies offer an opportunity to accurately recreate the heterogeneities of tumors, including their specific phenotypes and functions in vitro.^[^ ^119^, ^120^ ^]^ This section discusses the tumor microenvironment's capacity for establishing heterogeneous tumor models, as well as the manufacturing techniques developed for this purpose.

### 3.1 Regulation of Signal Expression for Engineering Heterogeneous Tumor Models

#### 3.1.1 VEGF‐related Vascular Reconstruction in Tumor Models

**Figure 6:**
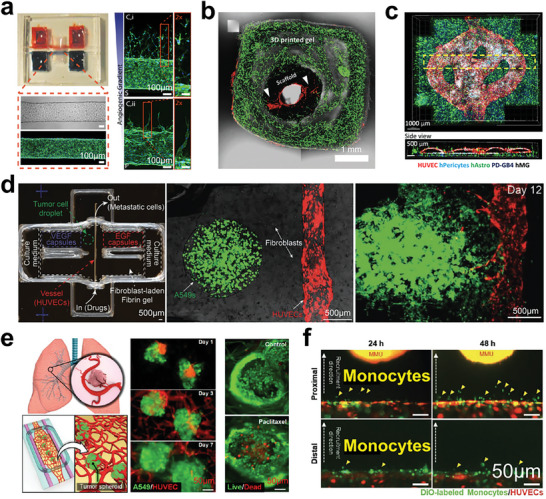
VEGF‐related vascular reconstruction in tumor models. a) The endothelialized channel in the microfluidic chip was exposed to the defined gradient of angiogenic factors. The vascular sprouting and migration can be visualized and quantified. Reproduced with permission,^[^ ^131^ ^]^ published by the National Academy of Science. b) A hollow and perfusable vascular construct was fabricated by printing a vascularized gel and endothelialized scaffold together. Reproduced with permission.^[^ ^132^ ^]^ Copyright 2021 Wiley‐VCH GmbH. c) Confocal images showing a printed glioblastoma model with hollow vascularized constructs, the dashed box is a cross‐section of the vessels. Reproduced with permission.^[^ ^133^ ^]^ Copyright 2021 the authors. d) A metastatic tumor model containing a vessel, fibroblasts, tumor cells, and VEGF signaling gradient. The A549 tumor cells can approach and enter the vasculature. Reproduced with permission.^[^ ^144^ ^]^ Copyright 2019 Wiley‐VCH Verlag GmbH & Co. KGaA, Weinheim. e) Vascularized lung tumor model in a microfluidic chip for testing the vascular toxicity of chemotherapy. The perfusable vascularized tumor model can be filled with a clinical dose of anti‐tumor drug (paclitaxel). Reproduced with permission.^[^ ^145^ ^]^ Copyright 2019 American Chemical Society. f) The tissue‐level cancer‐vascular model for replicating the tumor inflammatory process and quantitatively studying the monocyte recruitment. Reproduced with permission.^[^ ^123^ ^]^ Copyright 2021 Wiley‐VCH GmbH.

In the pursuit of “made‐to‐measure” tumor models, a comprehensive understanding and integration of the vasculature in tumor tissues is necessary.^[^ ^121^, ^122^, ^123^, ^124^, ^125^, ^126^, ^127^, ^128^ ^]^ The first event in vasculature growth is neovascularization, which is regulated by the VEGF events and essential in tumor development.^[^ ^129^, ^130^ ^]^ For instance, a team developed a microfluidic platform to quantitatively investigate angiogenic sprouting and neovessel formation (**Figure** **6**a).^[^ ^131^ ^]^ By using a biomimetic model, the authors demonstrated that endothelial cells invade as multicellular sprouts, which can be regulated by various proangiogenic factors such as bFGF, HGF, VEGF, and MCP‐1. Once vessels mature, they are capable of transporting nutrients and clearing waste. As an essential component of the tumor microenvironment, perfusable vascular constructs have been engineered in tumor models to study the circulatory system and extend the lifespan of models. In another way, Szklanny et al. printed hierarchical vascular constructs in vitro, enabling tissue perfusion through the scaffold lumen (Figure 6b).^[^ ^132^ ^]^ The inner endothelial cells can connect with the ECs in printed gel, creating a promising model with a functional vascular hierarchy. Recently, Neufeld et al. employed a bioprinting approach to engineer a perfusable glioblastoma model that recapitulated the tumor heterogenic microenvironment (Figure 6c).^[^ ^133^ ^]^ The printed glioblastoma model contained multiple cell components, such as HUVECs, hPericytes, PD‐GB4, and brain stromal cells (hAstro, hMG), and successfully formed vessel lumens that could transport nutrients and waste. Therefore, the vasculature fully participates in the VEGF signaling pathways and is a major component in tumor tissues, which is indispensable in the reconstruction of tumor heterogeneity.

Through the integration of matured vasculatures and other tumor components in vitro, processes such as metastasis, drug resistance, and inflammation during tumor development can be precisely studied.^[^ ^134^, ^135^, ^136^, ^137^, ^138^, ^139^, ^140^, ^141^, ^142^, ^143^ ^]^ Metastasis is regarded as the main reason for difficulty in tumor clearance, forming a new tumor site in other organs or tissues of the body. Along the metastatic process, the vascular network provides a route for tumor cell to travel in the body. In order to study tumor metastasis, Meng et al. described a vascularized tumor model to reconstruct tumor invasion, intravasation, and extravasation in vitro (Figure 6d).^[^ ^144^ ^]^ The authors achieved the quantitative interrogation of metastatic processes by the programmed release of growth factors. The incorporation of collection chambers into the system enabled the downstream analysis of CTCs that self‐select to intravasate. Recently, Paek et al. engineered a perfuable microvascular bed connected with multiple tumor spheroids in microfluidic chip.^[^ ^145^ ^]^ They incorporated the endothelial cells, lung fibroblasts, and cancer spheroids (lung carcinoma epithelial cells) to mimic malignant solid tumors in the lung. The vascularized tumor model was treated with a clinical antitumor drug (paclitaxel) for two days (Figure 6e). The tumor‐killing effect of this drug was quantified according to the overall viability of tumor cells. Particularly, paclitaxel‐induced vascular toxicities can be investigated, which is a promising therapeutic way to disrupt the vasculature and inhibit cancer angiogenesis. During tumor progression, endothelial cells can be activated by inflammatory cytokine signaling in tumor, resulting in the recruitment of circulating leukocytes like monocytes. Kim et al. simultaneously recapitulated EMT‐induced endothelial dynamics, and recruitment of immunocytes in vitro, enabling the understanding of the fundamentals of inflammatory responses between cancer and blood vessels (Figure 6f).^[^ ^123^ ^]^ Together, the vascular constructs can not only improve the robustness of tumor models but also extend the interrogation range of tumor homeostasis.

#### 3.1.2 Cadherin‐Related Cell Behaviors in Tumor Reconstruction

**Figure 7:**
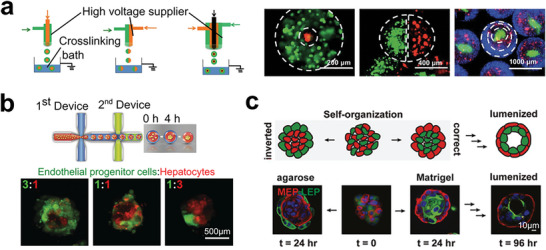
Cadherin‐related cell self‐organization in tumor reconstruction. a) A versatile multi‐fluidic electrostatic spraying technique for generating various cell encapsulations, enabling the self‐organization of three different layered cell microparticles. Reproduced with permission^[^ ^156^ ^]^ published by the Royal Society of Chemistry. b) A double‐emulsion microfluidics for generating cell spheroids with different cell organizations. Reproduced with permission.^[^ ^157^ ^]^ Copyright 2016 Wiley‐VCH Verlag GmbH & Co. KGaA, Weinheim. c) Self‐organization of initially disordered populations of cells into spatially ordered tissues under different cell‐ECM contact. Reproduced with permission,^[^ ^158^ ^]^ published by the National Academy of Science.

Cadherin is a crucial cell‐adhesion molecule that is essential in tumor morphogenesis, proliferation, and development.^[^ ^146^, ^147^, ^148^, ^149^, ^150^, ^151^, ^152^ ^]^ The ability of cadherin to maintain intracellular cohesion and preserve the architecture of tumor tissue offers opportunities for programmable and bottom‐up design of the tumor microenvironment.^[^ ^148^, ^153^, ^154^, ^155^ ^]^ The multicellular components that organize into a specific order are among the major features of tumor heterogeneity. To mimic this, Lu et al. used electrostatic spraying technique to fabricate uniform spheroids with controllable multicellular distributions (**Figure** **7**a).^[^ ^156^ ^]^ NHLFs, MCF‐10A, and MDA‐MB‐231 cells were encapsulated into different organizations for this purpose. To further explore the potential of tumor self‐organization, Chan et al. presented a double‐emulsion microfluidic technique for generating hepatocyte spheroids with controllable cell encapsulation (Figure 7b).^[^ ^157^ ^]^ They claimed that the expression of hepatocyte functions was improved after multicellular organization. Additionally, the self‐organization process can be controlled by the cell‐ECM boundary. Moreover, one work studied the influence of various ECMs on cell organization and found that sufficient cell‐ECM cohesion contributed to epithelial tissue polarization (Figure 7c).^[^ ^158^ ^]^ Thus, self‐organization in tumor tissue can be controlled by the expression and distribution of cadherin. These developments in understanding the role of cadherin in tumor self‐organization hold great potential for the design of innovative therapeutic strategies for cancer treatment.

**Figure 8:**
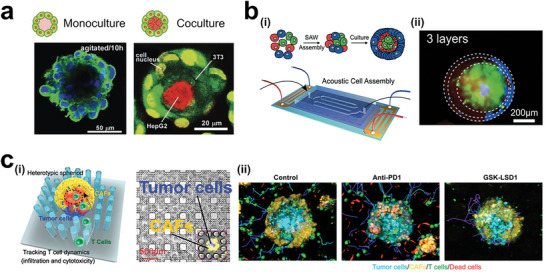
Cadherin‐related cell sorting in tumor reconstruction. a) Cell‐sorting of cells on the surface of collagen beads. In the monoculture, F‐actin (Alexa488‐conjugated phalloidin in green fluorescence), cell nuclei (Hoechst 33 342 in blue fluorescence). In the coculture, the NIH 3T3 cells covered the encapsulated HepG2 cells. Reproduced with permission.^[^ ^162^ ^]^ Copyright 2011 Wiley‐VCH Verlag GmbH & Co. KGaA, Weinheim. b) Cell layers achieved by the heterogeneous expression of cadherin proteins on acoustic‐assembly cell spheroids containing cancer cells (EO771 in green fluorescence), fibroblast (3T3 in blue fluorescence), Endothelial cells (2H11 in red fluorescence). Reproduced with permission.^[^ ^163^ ^]^ Copyright 2021 The Royal Society of Chemistry. c) Heterotypic spheroids with core/tumor‐shell/stroma cell distribution for investigating the process of immunocyte infiltration into solid tumors. i) Heterotypic tumor spheroids formed in pillar arrays. Tumor cells (Blue color); CAFs (Yellow color). ii) T cell infiltration inside tumor spheroids treated by immune‐related drugs. Reproduced with permission.^[^ ^164^ ^]^ Copyright 2022 the authors, under CC BY‐NC‐ND license.

During tumor development, the ability of multiple cell components to self‐organize into spatially ordered structures, known as cell sorting, plays a crucial role.^[^ ^159^, ^160^, ^161^ ^]^ Cadherin, as a cell‐adhesion molecule, determines cell–cell contacts and enables the formation of complex architectures. The type and amount of cadherin expression on the cell membrane can influence the organization of cells in developing tissues. To more realistically mimic tissues in vitro, Matsunaga et al. developed a microfluidic technique that generates monodisperse beads loaded with self‐organizing cells. Collagen gel beads seeded with HepG2 and NIH 3T3 cells can form hierarchic co‐cultures to mimic tissues more accurately (**Figure** **8**a).^[^ ^162^ ^]^ More recently, Wu et al. used an acoustic assembly approach to generate heterotypic cell spheroids in a scaffold‐free manner (Figure 8b).^[^ ^163^ ^]^ The authors aggregated three types of cells that expressed different cadherin molecules to reconstruct the spatial heterogeneity of tumor tissues. The cadherin‐driven cell sorting enabled the clear layering of three cell types within cell spheroids. Moreover, Ao et al. developed a pillar array chip to create uniform spheroids with a physical barrier, a tight stromal layer of CAFs on the tumor core. The layered spheroids replicated the immune‐cell infiltration process and killing events (Figure 8c).^[^ ^164^ ^]^ This platform provides a robust approach to investigate immune‐tissue interactions and is useful in immunology, oncology, tissue engineering, and precision medicine.

**Figure 9:**
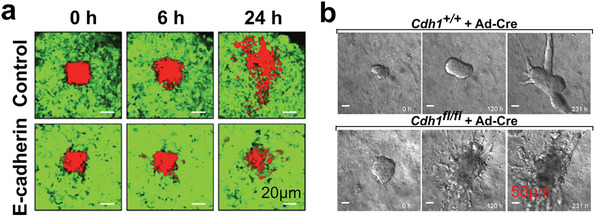
Cadherin‐related cell rearrangements in tumor reconstruction. a) Images showing the lower collective cell movement of GFP‐E‐cadherin‐expressing tumor cells compared to the control group. Reproduced with permission.^[^ ^170^ ^]^ Copyright 2010 American Association for Cancer Research. b) Time‐lapse images showing Cdh1^+/+^ and Cdh1^fl/fl^ organoids. Reproduced with permission.^[^ ^171^ ^]^ Copyright 2019 the authors, under exclusive license to Springer Nature Limited.

Cancer‐related deaths are mainly due to the metastatic potential of tumor tissues, which have the ability to rearrange themselves into the most aggressive modes and shapes.^[^ ^165^, ^166^ ^]^ Tumor cells employ diverse strategies to alter their form and migrate in vivo. E‐cadherin, an important suppressor of tumor invasion, can be controlled by tumor cells.^[^ ^167^, ^168^, ^169^ ^]^ To elucidate the role of E‐cadherin in tumor tissues, one study presented a tumor invasion model by embedding epidermoid carcinoma A431 cells (shown in red) into a collagen gel in vitro (**Figure** **9**a).^[^ ^170^ ^]^ The researchers found that overexpressed E‐cadherin (depicted in green) in tumor cells can impede cell motility, suggesting that tumor cells depend on E‐cadherin‐mediated cell‐cell adhesion during the movement process in vivo. Cell rearrangements can occur not only in the form of coordinated neighbor exchange but also lead to greater deformation and even dissemination. Interestingly, Padmanaban et al. proposed that the loss of E‐cadherin is a trigger for invasion and metastasis. The authors embedded cells with and without E‐cadherin expression into a 3D collagen I matrix and found that E‐cadherin‐negative cells showed lower migratory persistence and displacement, suggesting the importance of E‐cadherin in the growth, invasion, dissemination, and metastatic colonization of tumor tissue (Figure 9b).^[^ ^171^ ^]^ These results indicated that studying cadherin function in tumor tissues can aid in developing desired heterogeneous tumor models.

### 3.2 Regulation of Cell Proliferation for Engineering Heterogeneous Tumor Models

#### 3.2.1 CSCs‐Induced Tumor Self‐Organization

The initiation of new tumors may stem from a solitary tumor cell type known as cancer stem cells (CSCs), which can replicate numerous critical traits of the primary tumor tissues.^[^ ^172^, ^173^, ^174^, ^175^, ^176^, ^177^ ^]^ Recently, tumor organoids have emerged as a promising platform for modeling tumor heterogeneity in vitro.^[^ ^178^, ^179^, ^180^ ^]^ Tumor organoids represent assemblages of neoplastic cells obtained from human‐ and mouse‐related cancer tissues. These organoids effectively recapitulate the tumor microenvironment, enabling the study of tumor heterogeneity from fundamental research to translational research.^[^ ^181^, ^182^, ^183^, ^184^ ^]^

**Figure 10:**
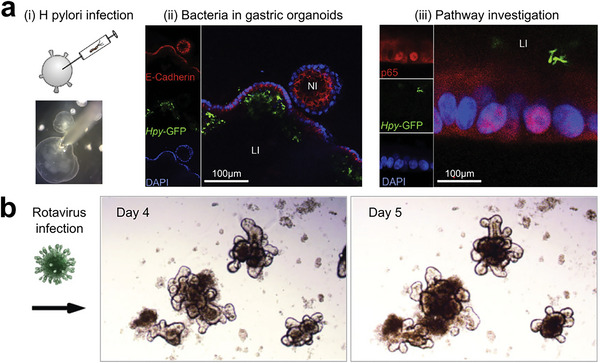
Organoids for infection research. a) Gastric organoids for studying bacteria infection. i) The working flow of injecting *H. pylori* into an organoid; ii) Fluorescent images show a lumen‐infected (LI) organoid and a noninfected (NI) organoid. Bacteria were visualized using GFP expression, organoids were stained with E‐cadherin (red) and DAPI (blue); iii) The investigation of the bacteria‐related pathway (NF‐kB subunit p65). Reproduced with permission.^[^ ^191^ ^]^ Copyright 2015 the AGA Institute, under CC BY‐NC‐ND license. b) Modeling rotavirus infection using intestinal organoids. Reproduced with permission.^[^ ^192^ ^]^ Copyright 2015 Elsevier.

Researchers have taken a keen interest in studying organoids cultured with pathogens with the potential to induce cancer diseases.^[^ ^185^, ^186^, ^187^, ^188^, ^189^, ^190^ ^]^ For instance, chronic infection is known to cause gastric cancer. The Netherlands‐based team first employed a gastric culture platform, allowing for the long‐term (>1 year) culturing of gastric cells (**Figure** **10**a).^[^ ^191^ ^]^ These organoid cultures can be leveraged to investigate stem cell biology and the response of the epithelium to infection. Notably, the team found that the nuclear factor‐kB (NF‐kB) pathway was activated after only 1 h of H pylori infection in organoids. Meanwhile, Yin et al. established intestinal organoid cultures for reconstructing rotavirus infection (Figure 10b).^[^ ^192^ ^]^ They introduced rotavirus SA11 into organoid cultures on day 4 and found viral infection phenomena after 24 h through a positive immunohistochemical process on viral protein VP4. Thus, stem cell‐derived organoids give a great chance for investigating the relationship between infectious agents and cancer development, and exploring the potential antiviral therapy.

**Figure 11:**
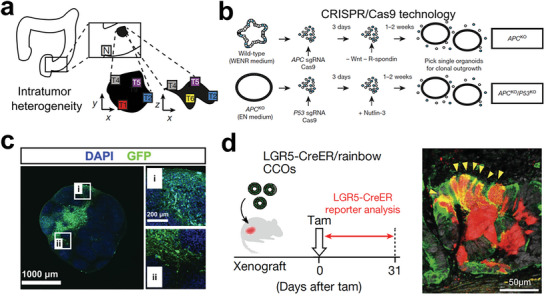
Organoids for genetic and stem cell research. a) Studying the nature of diversification in tumor tissues by characterizing the organoid cultures that derived from cancer sites and adjacent normal intestinal crypts. Reproduced with permission.^[^ ^199^ ^]^ Copyright 2018 Macmillan Publishers Limited, part of Springer Nature. b) CRISPR/Cas9 method used in gene modification of four major mutated colorectal cancer genes in stem cells. Stem cells (blue); EGF (E); Noggin (N); R‐spondin (R); WNT (W). Reproduced with permission.^[^ ^201^ ^]^ Copyright 2015 Macmillan Publishers Limited. c) Cerebral organoids were genetically engineered to form brain tumors. Fluorescent images showing the tumour‐normal interface in GBM‐1 neoplastic cerebral organoid. Reproduced with permission.^[^ ^202^ ^]^ Copyright 2018 the authors. d) The lineage‐tracing strategy of LGR5^+^ cells. Fluorescent image showing the immunostaining of KRT20 (green) and RFP (red). Reproduced with permission.^[^ ^203^ ^]^ Copyright 2017 Macmillan Publishers Limited, part of Springer Nature.

Organoid cultures have emerged as a powerful tool for investigating various aspects of cancer development, including mutagenic processes and tumor heterogeneity.^[^ ^193^, ^194^, ^195^, ^196^, ^197^, ^198^ ^]^ Their high genetic stability over long periods enables the detailed study of mutational patterns, as demonstrated by Roerink et al. in their investigation of intratumour heterogeneity of colorectal cancers (**Figure** **11**a).^[^ ^199^ ^]^ Moreover, organoids can model and study cancer initiation and progression^[^ ^200^ ^]^, as shown by Drost et al., who utilized CRISPR/Cas9 to edit four cancer genes in stem cells (Figure 11b).^[^ ^201^ ^]^ Bian et al. further recapitulated brain tumorigenesis in brain organoids by using gene‐editing technique (Figure 11c).^[^ ^202^ ^]^ However, identifying CSC development in tumor tissues remains a challenge. Shimokawa et al. overcame this obstacle by revealing the developmental ability of LGR5^+^ tumor cells by using lineage‐tracing experiments (Figure 11d).^[^ ^203^ ^]^ Overall, CSC‐induced tumor organoids represent robust models for basic cancer research and have great potential for further advancement.^[^ ^202^, ^204^, ^205^, ^206^, ^207^ ^]^

**Figure 12:**
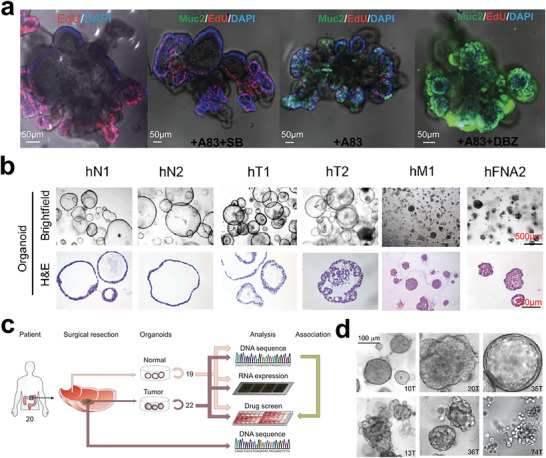
Organoids for translational research. a) The development of human organoid cultures can be well regulated by changing the growth factors in the culture medium. Reproduced with permission.^[^ ^217^ ^]^ Copyright 2011 the AGA Institute. b) Pancreatic ductal organoids derived from different tumor regions of cancer patients. Reproduced with permission.^[^ ^218^ ^]^ Copyright 2015 Elsevier Inc. c) Human colorectal normal and tumor organoids used in drug screening for identifying gene‐drug associations. Reproduced with permission.^[^ ^219^ ^]^ Copyright 2015 Elsevier Inc. d) Living biobank of breast cancer organoids can capture original tumor heterogeneity. Reproduced with permission.^[^ ^220^ ^]^ Copyright 2017 Elsevier Inc.

Tumor heterogeneity poses a significant challenge in cancer research and personalized therapy.^[^ ^208^, ^209^, ^210^ ^]^ In recent years, the development of CSC‐derived tumor organoids has become a promising approach to recapitulate the complexity and diversity of tumors in vitro.^[^ ^211^, ^212^, ^213^, ^214^, ^215^, ^216^ ^]^ Notably, the pioneering work by Sato et al. in 2009 demonstrated the formation of 3D organoid cultures based on single LGR5^+^ intestinal stem cells, providing a platform to investigate stem cell biology and intestinal diseases (**Figure** **12**a).^[^ ^217^ ^]^ Since then, several CSC‐derived tumor organoids have been established, including pancreatic organoids by Boj et al., which recapitulated normal and neoplastic ducts in patient samples (Figure 12b).^[^ ^218^ ^]^ Furthermore, living organoid biobanks, such as the colorectal cancer biobank by Wetering et al.^[^ ^219^ ^]^ and the breast cancer biobank by Sachs et al.^[^ ^220^ ^]^ was developed to enable high‐throughput drug screening and personalized therapy design (Figure 12c,d). These advances can provide a deeper understanding of tumor biology, and have the potential to transform cancer therapy as well.

**Figure 13:**
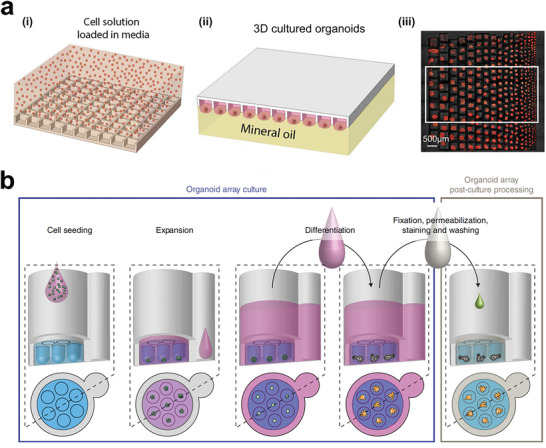
Advances in tumor organoid engineering for translational research. a) Hanging drop culture allows high replicates of tumor organoids for on‐chip confocal microscopy. i) Cell loading in microarray chips. ii) Chip inverted for cell culture. iii) Typical image showing tumor spheroids formation in microarray chip with gradient sizes. Reproduced with permission.^[^ ^229^ ^]^ Copyright 2021 the authors. b) Microcavity arrays for high‐throughput automated organoid culture and real‐time analysis of anticancer drug's efficacy. Reproduced with permission.^[^ ^230^ ^]^ Copyright 2020 the authors, under exclusive license to Springer Nature Limited.

Organoid culture systems have attracted widespread attention for their ability to recapitulate the complexity of in vivo tissue in vitro. However, the reproducibility and scalability of organoid culture systems remain a significant challenge for their wider use in biomedical research and drug discovery.^[^ ^221^, ^222^, ^223^, ^224^, ^225^, ^226^ ^]^ To overcome this limitation, engineers have developed various approaches to optimize organoid culture, including the development of microchip hanging drop platforms and automated culture systems.^[^ ^227^, ^228^ ^]^ Ganguli et al. developed a modular microchip platform with silicon microwells of varying dimensions for tumor organoid culture, enabling high‐throughput drug screening and geometric control applications (**Figure** **13**a).^[^ ^229^ ^]^ Through single‐cell RNA sequencing, the authors demonstrated the high degree of genomic similarity between patient‐derived xenografts (PDX) and 3D organoid cultures. Similarly, Brandenberg et al. developed an automated organoid culture system with high‐throughput performance for testing patient‐derived colorectal cancer organoids with drugs in FDA‐approved or clinical trials (Figure 13b).^[^ ^230^ ^]^ The use of organoids has shown promising results in replicating the heterogeneity of drug responses in tumors, suggesting the potential of organoid culture systems in personalized medicine.

#### 3.2.2 Tumor Models Preserve Primary Cell Components

**Figure 14:**
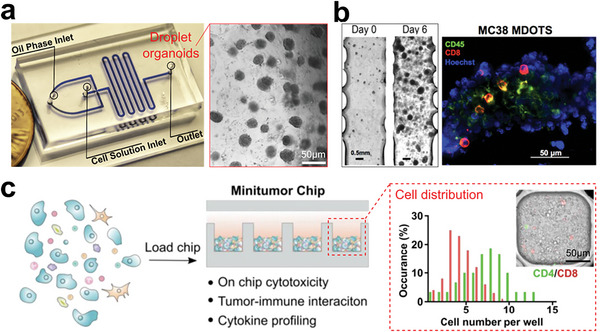
Tumor models preserve all the cell components including immune cells without primary tumor structures. a) Droplet microfluidics for producing uniform patient‐derived tumor clusters. Reproduced with permission.^[^ ^235^ ^]^ Copyright 2020 American Chemical Society. b) Ex vivo system for culturing murine‐ and patient‐derived organotypic tumor spheroids (MDOTS/PDOTS) that can model the responses to immune checkpoint blockade (ICB). Reproduced with permission.^[^ ^236^ ^]^ Copyright 2018 American Association for Cancer Research. c) Mini‐tumor chips for evenly distributing dissociated tumor cells and reserving tumor immune microenvironment. Reproduced with permission.^[^ ^237^ ^]^ Copyright 2023 Ivyspring International Publisher.

In the realm of heterogeneous tumor models, preserving the primary tumor cell components is of utmost importance. Such models facilitate the decoding of fundamental processes, even in the absence of the primary tumor structures.^[^ ^231^, ^232^, ^233^, ^234^ ^]^ Tumors are composed of heterogeneous cell populations with different genetic mutations and phenotypes. Tissue‐engineered models can incorporate various cell types and genetic alterations to mimic this heterogeneity. By manipulating the composition of cells in different regions of the model, researchers can observe how different subpopulations compete, evolve, and interact over time. Wu et al. recently developed a droplet microfluidics approach to fabricate 3D tumor spheroids comprising patient‐derived tumor cells, which were shown to maintain genetic features of primary tumor tissues and display various responses to chemotherapies in 48 h (**Figure** **14**a).^[^ ^235^ ^]^ Incorporating the tumor immune microenvironment into sophisticated tumor models has been crucial for studying anti‐tumor immune responses in patients. To this end, Jenkins et al. described a novel platform for fabricating organotypic tumor spheroids, allowing for the evaluation of tumor‐immune interactions (Figure 14b).^[^ ^236^ ^]^ This work has paved the way for developing novel therapeutic combinations and facilitating precision immune‐oncology efforts. More recently, Ao et al. presented the preclinical concept of mini‐tumor chip, which can evaluate the responses to cancer immunotherapy in a microfluidic chip.^[^ ^237^ ^]^ Via evenly suspending dissociated tumor cells into the microfluidic chip, mini‐tumors were formed on chip, offering an easy, quick‐turnaround solution to measure immunotherapy responses (Figure 14c).

**Figure 15:**
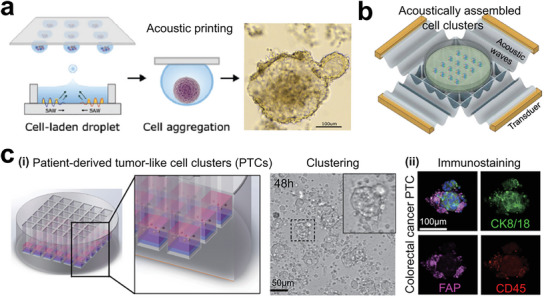
Tumor models preserve all the cell components without primary tumor structures for studying tumor immune microenvironment. a) Acoustic printing of microdroplets for establishing immunologic tumor organoids derived from patient tumor tissues. Reproduced with permission.^[^ ^245^ ^]^ Copyright 2021 Wiley‐VCH GmbH. b) Acoustic assembly of patient tumor cells for studying tumor immune microenvironment. Reproduced with permission.^[^ ^246^ ^]^ Copyright 2022 the authors. c) Patient‐derived tumor‐like cell clusters (PTCs) for maintaining the proliferation of primary cells in tumor tissues. i) Formation of PTCs in microarrays. ii) Immunostaining images showing the preserved cell components. Reproduced with permission.^[^ ^247^ ^]^ Copyright 2020 the authors, under the exclusive license American Association for the Advancement of Science.

Understanding the roles of immune components in solid tumors has become an area of intense interest, and various approaches have been developed to preserve immune components in close‐packed tumor models.^[^ ^238^, ^239^, ^240^, ^241^, ^242^, ^243^, ^244^ ^]^ Gong et al.^[^ ^245^ ^]^ and Ao et al.^[^ ^246^ ^]^ have utilized acoustic methods to accelerate cell contact and fabricate tumor organoids or clusters containing immune cells (**Figure** **15**a,b). The use of acoustic forces offers several advantages over other passive methods, including high biocompatibility and rapid aggregation of cells. However, it is worth noting that tight cell contact in tumor models can lead to hypoxia and nutrient shortage, which can negatively impact cell proliferation. In this case, Yin et al. showed a tumor cell cluster model derived from patients that enables everlasting culture and tumor cell's development under the non‐Matrigel medium (Figure 15c), thus improving cell viability.^[^ ^247^ ^]^ Overall, the development of various approaches for optimizing cell proliferation in tumor models is a rapidly evolving field.

**Figure 16:**
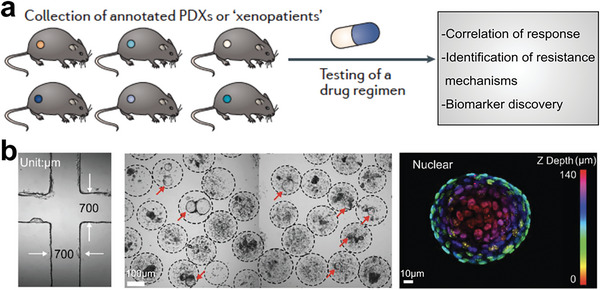
Tumor models reserve all the cell components with primary tumor structures. a) PDXs for addressing the patterns of cancer evolutionary dynamics during tumor progression. Reproduced with permission.^[^ ^248^ ^]^ Copyright 2017 Macmillan Publishers Limited, part of Springer Nature. b) Tumor organoids within the alginate microbeads develop luminal‐ and soild‐like structures. Reproduced with permission.^[^ ^264^ ^]^ Copyright 2021, the authors.

To achieve more reliable tumor models, several advanced approaches have been developed to retain both cell components and tumor structures. Byrne and colleagues have concluded that PDX models, defined as the culture of human tumor tissues in mice (**Figure** **16**a), can balance the tumor structures and cell components, thereby recapitulating intra‐ and inter‐tumor heterogeneity.^[^ ^248^, ^249^, ^250^, ^251^, ^252^, ^253^, ^254^, ^255^, ^256^ ^]^ A major obstacle in PDX models is the necessity of using immunocompromised mice to circumvent xenograft rejection, which hampers immune assessments.^[^ ^257^, ^258^, ^259^ ^]^ To overcome this limitation, researchers have attempted to use in vitro culture systems to replace animal hosts such as mice.^[^ ^260^, ^261^, ^262^, ^263^ ^]^ Recently, Fang et al. cultured organoids with tumor pieces in alginate microbeads, generating luminally structured tumor organoids that displayed high similarity to primary tumor tissues in cell phenotypes and lineages (Figure 16b).^[^ ^264^ ^]^

**Figure 17:**
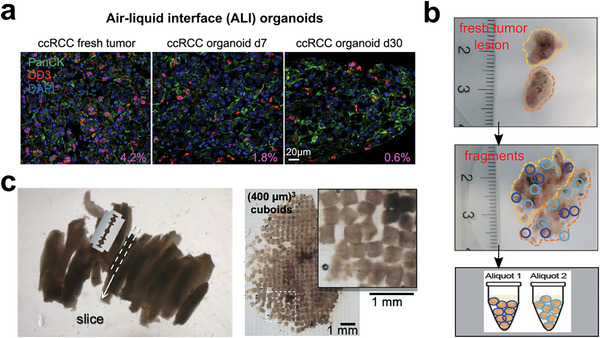
In vitro tumor cultures directly reserve all the cell components with primary tumor structures. a) Air‐liquid interface (ALI) organoid for reconstructing tumor immune microenvironment. % area ratio of CD3^+^ cells (red color). Reproduced with permission.^[^ ^265^ ^]^ Copyright 2018 Elsevier Inc. b) Patient‐derived tumor fragment platform (PDTF) that responds to immune drug treatment. Reproduced with permission.^[^ ^266^ ^]^ Copyright 2021 the authors, under the exclusive license to Springer Nature America, Inc. c) Cuboidal‐shaped microdissected tissues or “cuboids” preserving of tumor tissue microenvironment. Reproduced with permission.^[^ ^267^ ^]^ Copyright 2020 The Royal Society of Chemistry.

To increase the number of cell components and extend the culture time, Neal et al. established the air‐liquid interface (ALI) system for improving nutrient delivery and gas perfusion in living tumor sections (**Figure** **17**a).^[^ ^265^ ^]^ ALI patient‐derived tumor organoids can maintain the normal activities of stromal cells and immune cells for up to one month, and the authors demonstrated the recapitulation of tumor‐immune microenvironment using their tumor models. Voabil et al. used the ex‐vivo tumor fragment platform for preserving tumor immune microenvironment and architecture, as well as T cell‐related cytokines (Figure 17b).^[^ ^266^ ^]^ This pioneering work demonstrated the correlation between early immunological alterations and clinical response after PD‐1 blockade. Technically, Horowitz et al. developed a tumor model called cuboids, which were sectioned from patient tumor tissue into cuboidal shapes (Figure 17c).^[^ ^267^ ^]^ Together, these advanced engineering methods have enabled the establishment of tumor models with complete cell components and structures, offering valuable tools for metronomic and chronic drug assessments.^[^ ^268^, ^269^, ^270^, ^271^, ^272^, ^273^, ^274^ ^]^

#### 3.2.3 Tumor Models with Controllable Cell Organization

**Figure 18:**
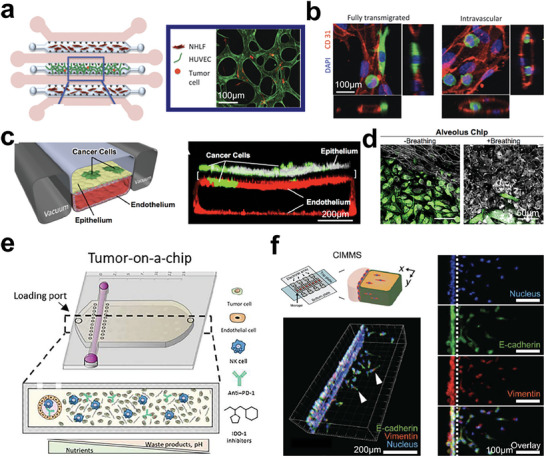
Microfluidic chips for hierarchy tumor engineering. a) In vitro microvascular networks for assessing tumor cell extravasation. b) Representative filed of live microvascular networks (HUVEC in red color) at 12 h after seeding tumor cells (MDA‐MB 231 in green color). Immunostaining of CD31 showed heterogeneity of transmigrated, and nontransmigrated cells. Reproduced with permission.^[^ ^290^ ^]^ Copyright 2018 American Association for Cancer Research. c) Organ‐on‐chip modeling of orthotopic lung cancer growth under breathing motions. d) Lung cancer cells (green color) displayed different development statuses in a normal epithelial monolayers after 14 days of culture in the airway chip with and without breathing motions. Reproduced with permission.^[^ ^291^ ^]^ Copyright 2018 the authors, under the CC BY‐NC‐ND license. e) Microfluidic chip for replicating tumor‐induced suppressive environment and evaluating NK cell exhaustion. Reproduced with permission.^[^ ^292^ ^]^ Copyright 2021 the authors, under the exclusive license to American Association for the Advancement of Science. f) Cell invasion in digital microfluidic microgel systems (CIMMS) corporated with basement membrane and tumor cells. Reproduced with permission.^[^ ^293^ ^]^ Copyright 2020 the authors, under the exclusive license to American Association for the Advancement of Science.

So far, advancements in tumor models have enabled the attainment of controllable cell distributions. Among emerging technologies, microfluidics holds enormous potential for precise control over cell behavior and organization at the microscale level.^[^ ^275^, ^276^, ^277^, ^278^, ^279^, ^280^, ^281^, ^282^, ^283^, ^284^, ^285^, ^286^ ^]^ In tumor tissue engineering, microfluidic chips have been employed for arranging vascular networks in collaboration with tumor cells.^[^ ^287^, ^288^, ^289^ ^]^ With high spatiotemporal resolution, Hassell et al. studied the morphology of transmigrating tumor cells and related endothelial cells in vitro (**Figure** **18**a,b).^[^ ^290^ ^]^ Notably, one work incorporated physiological breathing motions into their lung cancer chip, mimicking the dynamic structure and function of the lung, which resulted in significant suppression of lung cancer growth (Figure 18c).^[^ ^291^ ^]^ Without the breathing motions, tumor cells tended to grow into larger areas in the epithelium layer (Figure 18d). Moreover, a team presented a tumor‐chip model. This model allowed to arrange tumor cells and endothelial cells inside a 3D matrix (Figure 18e).^[^ ^292^ ^]^ Their model enabled the recapitulation of nutrient gradient within solid tumors, facilitating the exploration of NK cells’ capacity and functionality in activating immune processes. In addition, the ECM in tumors is often altered and can influence tumor behavior and evolution. Tissue‐engineered models can incorporate ECM components that mimic the tumor microenvironment, allowing researchers to investigate how ECM properties affect tumor progression and response to therapies. Li et al. showed a digital microfluidics system that successfully modeled cancer cell invasion and the analysis of RNA‐seq (Figure 18f).^[^ ^293^ ^]^ Collectively, microfluidic techniques provide a promising approach for controlling the locations of cells and even the matrix originating from tumor tissues.

**Figure 19:**
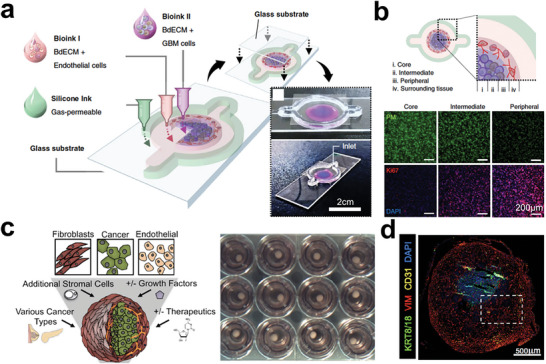
Bioprinting techniques for engineering functional and structural tumor tissues. a) Glioblastoma (GBM)‐on‐a‐chip was printed by using bioinks and various cells to construct a compartmentalized structure. b) Printed glioblastoma model with four layers mimicking primary tumor microenvironment. Reproduced with permission.^[^ ^304^ ^]^ Copyright 2019 the authors, under the exclusive license to Springer Nature Limited. c) 3D‐printed tumor constructs including multiple cell types in a defined spatial architecture. d) Immunofluorescence of the printed tissue sections. Vimentin (VIM, red color); KRT8/18 (green color); CD31 (yellow color); DAPI (blue color). Reproduced with permission.^[^ ^51^ ^]^ Copyright 2019 the authors.

Despite the precision afforded by microfluidics‐based tumor models in manipulating tumor cells during events like tumor angiogenesis, they presented challenges in constructing complex cancer systems that involve multiple interactions between cells, extracellular matrix (ECM), and physiological factors. Bioprinting, on the other hand, offers a direct method for generating 3D tissues containing various cell types in a defined spatial architecture.^[^ ^294^, ^295^, ^296^, ^297^, ^298^, ^299^, ^300^, ^301^, ^302^, ^303^ ^]^ Recently, Yi et al. demonstrated the ability to print multilayered glioblastoma tumor models using biocompatible bioinks and cells, including patient‐derived tumor cells and vascular endothelial cells (**Figure** **19**a).^[^ ^304^ ^]^ The GBM‐on‐chip model comprised four layers: the “core,” “intermediate,” “peripheral,” and surrounding tissue (Figure 19b). Similarly, one work tried to use 3D bioprinting to integrate various cell types, such as patient‐derived breast or pancreatic tumor cells, achieving the scaffold‐free building performance in tumor models (Figure 19c).^[^ ^51^ ^]^ Under the maturation of tissues, cells can deposit ECM and are self‐organized, exposing cancer cells to growth factors from different types of cells (Figure 19d).

**Figure 20:**
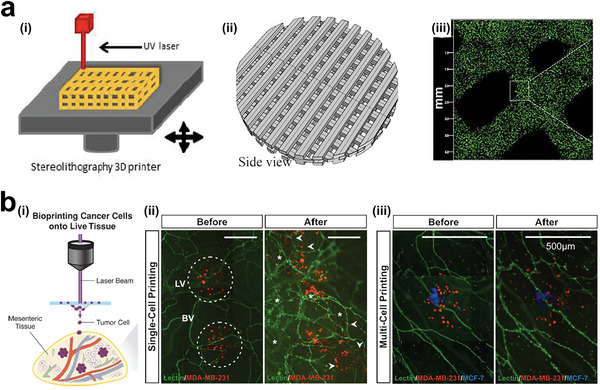
Bioprinting techniques for engineering functional and structural tumor tissues. a) 3D printing (i) of cell‐laden bone matrix (ii) including osteoblasts/MSCs embedded in GelMA and nHA hydrogel, which was co‐cultured with breast tumor cells (iii). Reproduced with permission.^[^ ^305^ ^]^ Copyright 2016 American Chemical Society. b) Laser direct‐write technique to print tumor onto living tissues (i). Fluorescence images showing the tumor cell location at day 0 and day 5 inside rat mesenteric tissue (ii, iii). “BV” represents blood vessel; “LV” represents lymphatic vessel. Reproduced with permission.^[^ ^306^ ^]^ Copyright 2016 Wiley Periodicals, Inc.

In addition, Zhou et al. employed a 3D bioprinting technique to develop the biomimetic bone matrix for investigating the crosstalking between diverse cell types in tumor tissue, creating a bone‐like microenvironment that facilitated the integration of all cell (**Figure** **20**a).^[^ ^305^ ^]^ Additionally, Burks et al. directly built tumor tissue onto rat living tissues based on laser direct‐write techniques, enabling real‐time studying of tumor cell migration, proliferation, and behaviors in angiogenesis (Figure 20b).^[^ ^306^ ^]^ In conclusion, bioprinting techniques offer a valuable tool for bridging the gap between in‐vivo tumor tissues and in‐vitro tumor models, facilitating the study of complex cancer systems.

**Figure 21:**
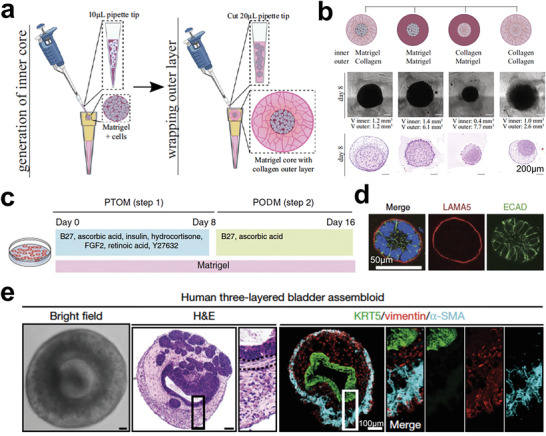
Tumor organoids with polarized cell structures. a) Oil‐in‐water droplet approach can fabricate uniform, microscale, two‐compartment organoids. b) Representative bright‐filed and H&E images showing the breast tumor organoids with two‐cell‐compartment inside after 7‐day culture by using different matrices. Reproduced with permission.^[^ ^319^ ^]^ Copyright 2022 Elsevier. c) Schematics of inducing progenitor orgnaoids into ductal and acinar cells. Pancreatic progenitor and tumor organoid medium (PTOM); Pancreatic organoid differentiation medium (PODM). d) Representative fluorescence images showing organoids with basal marker laminin‐α5 (LAMA5, red) and E‐cadherin (ECAD, green). Reproduced with permission.^[^ ^320^ ^]^ Copyright 2015 Nature Publishing Group, a division of Macmillan Publishers Limited. e) Human three‐layered bladder assembloids with tissue stroma‐stromal fibroblasts, endothelial cells, and a muscle layer. Reproduced with permission.^[^ ^321^ ^]^ Copyright 2020 the authors, under the exclusive license to Springer Nature Limited.

In last two decades, organoid technique has significantly advanced, enabling the modeling of key features in tissues and organs, facilitating a deeper understanding of different aspects of human cancer diseases.^[^ ^307^, ^308^, ^309^ ^]^ However, uncontrollable cell proliferation within tumor organoids commenly results in the loss of native tissue architecture and microenvironment, which has prompted the development of several engineering techniques to regulate cell proliferation within organoids.^[^ ^310^, ^311^, ^312^, ^313^, ^314^, ^315^, ^316^, ^317^, ^318^ ^]^ For instance, Lee et al. employed a simple oil‐in‐water droplet technology to uniformly fabricate multi‐compartment organoids in a high‐throughput manner (**Figure** **21**a).^[^ ^319^ ^]^ The authors demonstrated how matrix components influence tumor organoids growth, faithfully recapitulating tumor development monitored in vivo: tumor cells initially grew within a confined microenvironment before migrating to the collagen‐rich matrix regions (Figure 21b). To induce more functions inside tumor organoids, surrounding tissues can also be incorporated. Recently, one work developed a culture system to establish exocrine progenitor organoids with specific architectures (Figure 21c,d).^[^ ^320^ ^]^ Additionally, Kim et al. presented an impressive work that created multilayer bladder “assembloids” derived from human cells. They reconstituted tissue stem cells into an organized architecture with stromal components surrounded by epithelium layer, and a muscle layer outside (Figure 21e).^[^ ^321^ ^]^ These assembloids were able to exhibit key features of mature adult bladders. The authors demonstrated that the recapitulation of cell composition and gene expression. In particular, they recapitulated in vivo tissue kinetics of regenerative response to injury conditions. Together, tumor organoids with polarized cell distributions offer researchers significant opportunities to construct tumor tissues in a bottom‐up way.

### 3.3 Multidimensional Control of Tumor Structures

#### 3.3.1 Control of Tumor Model's Structures in 1D and 2D

**Figure 22:**
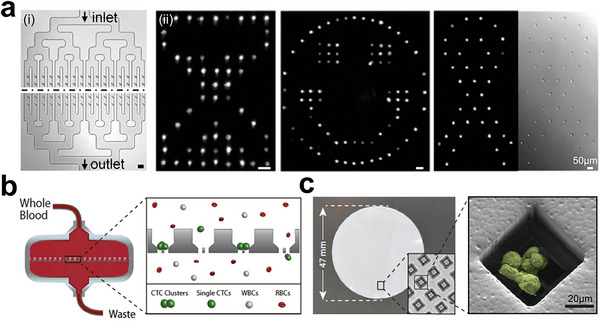
Tumor models with controllable 2D structures at single‐cell level. a) Microfluidics‐based living‐cell printing of functional single‐cell arrays. Reproduced with permission.^[^ ^335^ ^]^ Copyright 2014 National Academic of Science. b) Circulating tumor cells (CTCs) were isolated from the whole blood by using microfluidic chips. c) CTC clusters were formed within microwells. Reproduced with permission.^[^ ^337^ ^]^ Copyright 2022 the authors.

In the field of precision oncology, 1D and 2D tumor engineering have been widely employed to study cell‐cell communication, rare cell profiling, and tissue engineering.^[^ ^322^, ^323^, ^324^, ^325^, ^326^, ^327^, ^328^, ^329^, ^330^, ^331^, ^332^, ^333^, ^334^ ^]^ Zhang and colleagues introduced a microfluidic array of hook‐shaped traps for holding cells at designated positions (**Figure** **22**a).^[^ ^335^ ^]^ This “Block‐cell‐printing” platform was employed for quantitative analysis of tumor cell spreading in response to growth factor concentration. In addition to cell behavior studies, profiling of molecular information of tumor cells such as circulating tumor cells (CTCs) has been used in cancer diagnosis and management.​^[^ ^336^ ^]^ Boya and coworkers developed a device to isolate CTCs from whole blood and established a CTC cluster model (Figure 22b, c).^[^ ^337^ ^]^ By using RNA sequencing, the authors analyzed a subset of CTC clusters ranging from 2 to 100 cells.

**Figure 23:**
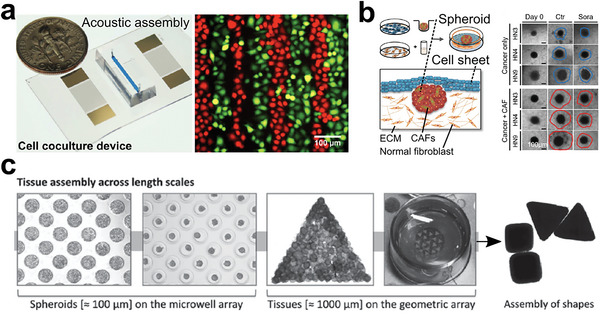
Tumor models with controllable 2D structures of cell lines and cell sheets. a) Acoustic assembly of cell lines with different cell types in a precise manner. Reproduced with permission.^[^ ^338^ ^]^ Copyright 2014 American Chemical Society. b) In vitro cell‐sheet cancer model containing a cancer spheroid and an oral mucosal cell sheet. Reproduced with permission.^[^ ^342^ ^]^ Copyright 2018 Ivyspring International Publisher. c) Microwell arrays for bottom‐up assembling of heterogeneous tissues. Reproduced with permission.^[^ ^141^ ^]^ Copyright 2016 Wiley‐VCH GmbH & Co. KGaA, Weinheim.

In 1D tumor engineering, surface acoustic waves were utilized by Li and colleagues to precisely pattern two types of tumor cells (**Figure** **23**a).^[^ ^338^ ^]^ The authors quantitatively analyzed tumor cell movements in a microfluidic chamber. In 2D tumor engineering, precise control of tumor tissue structure can provide characteristics closer to those of tumors in vivo.^[^ ^339^, ^340^, ^341^ ^]^ Lee et al. recently developed an epithelial cancer model comprising a cell sheet and spheroids to mimic the tumor microenvironment (Figure 23b).^[^ ^342^ ^]^ The cell sheet was composed of keratinocytes and fibroblasts, while cell spheroids were made up of cancer cells and cancer‐associated fibroblasts (CAFs). This model facilitated easy observation of drug resistance phenomena. Cell sheet, which has more mature structures and tighter cell‐cell contact than single cell‐based tumor engineering, can be assembled into material‐free tissues with complex architectures using an approach presented by Vrij and colleagues (Figure 23c).^[^ ^141^ ^]^ In conclusion, both 1D and 2D approaches to tumor engineering have unique features that can be employed to answer specific scientific questions.^[^ ^43^, ^343^, ^344^, ^345^ ^]^

#### 3.3.2 Control of Tumor Model's Structures in 3D

**Figure 24:**
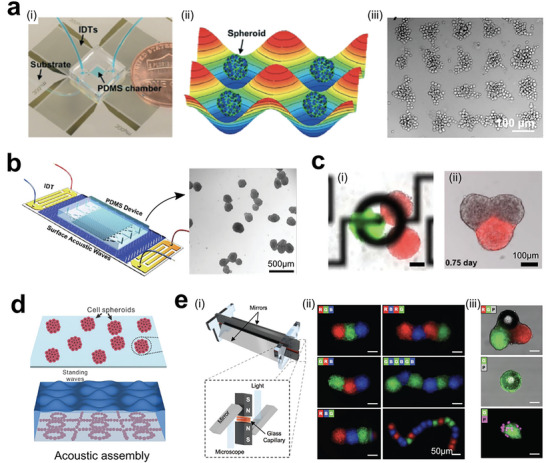
3D Tumor models fabricated from cell spheroid or organoid blocks. a) Acoustic assembly of tumor spheroids that can be patterned into grid‐like arrays. i) Acoustic device; ii) Acosutic pressure distribution for assembling cells; iii) Cell spheroids formation in grid‐like patterns. Reproduced with permission.^[^ ^357^ ^]^ Copyright 2016 The Royal of Society of Chemistry. b) Microfluidics‐assisted acoustic assembly of multicellular spheroids in a high‐throughput manner. Reproduced with permission.^[^ ^358^ ^]^ Copyright 2019 The Royal of Society of Chemistry. c) Acoustofluidic arrangement i) of tumor spheroids that fused into a big cell spheroid ii). Reproduced with permission.^[^ ^359^ ^]^ Copyright 2020 IOP Publishing Ltd. d) Biotunable acoustically assembling of cell spheroids and organoids. Reproduced with permission.^[^ ^360^ ^]^ Copyright 2015 Wiley‐VCH Verlag GmbH & Co. KGaA, Weinheim. e) Magnetic‐assisted assembly (i) and coding of 3D living architectures into designated geometry and organization (ii, iii). Reproduced with permission.^[^ ^361^ ^]^ Copyright 2017 Wiley‐VCH Verlag GmbH & Co. KGaA, Weinheim.

To achieve cell density and tumor structures that mimic the natural extracellular matrix (ECM), cell spheroids and organoids densely packed with living cells have been developed as a 3D microenvironment.^[^ ^346^, ^347^, ^348^, ^349^, ^350^, ^351^, ^352^, ^353^ ^]^ Various engineering techniques are employed for arranging cell spheroids or organoids, achieving the establishment of well‐designed architectures.^[^ ^354^, ^355^, ^356^ ^]^ For instance, 3D spheroids and organoids can be precisely patterned into 2D arrays. Chen and colleagues presented an approach to fabricate uniform tumor spheroids inside a microfluidic chip using surface acoustic waves (**Figure** **24**a), demonstrating that these spheroids can be applied in drug testing.^[^ ^357^ ^]^ To improve the acoustic assembly approach, Chen et al. integrated a Polydimethylsiloxane (PDMS) chip with multiple channels, scaling up the productivity of tumor spheroids (Figure 24b).^[^ ^358^ ^]^ On‐chip control systems are more advantageous for manipulating cell spheroids or organoids because they are scaffold‐free, versatile, and easy‐to‐culture in situ. Cai and colleagues showed a novel platform based on a C‐shaped surface acoustic wave (SAW) generator to manipulate cell spheroids and organoids using localized acoustic streaming (Figure 24c).^[^ ^359^ ^]^ They also quantitatively studied the fusion process of multiple spheroids and even brain organoids. Recently, Chen et al. used a powerful and tunable acoustic technology to assemble organoids into acoustic pressure nodes (Figure 24d).^[^ ^360^ ^]^ The authors successfully generated various patterns of spheroids and organoids, allowing to study the maturation process of in vitro tissue constructs. To achieve a higher degree of manipulation, Tocchio et al. developed a magnetic platform to assemble 3D tumor spheroids and arrange them into complex structures (Figure 24e).^[^ ^361^ ^]^ Cell spheroids can be arranged into desired patterns in a programmable way.

**Figure 25:**
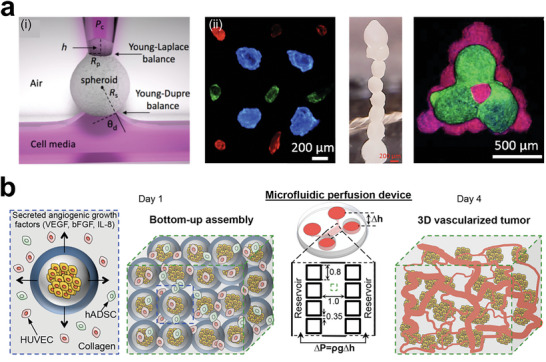
Complex 3D Tumor models fabricated from cell spheroid or organoid blocks. a) Aspiraion‐assisted bioprinting (i) of multicellular spheroids into complex structures (ii). Reproduced with permission.^[^ ^371^ ^]^ Copyright 2020 the authors, under the exclusive license to American Association for the Advancement of Science. b) Microtumors assembled within a micochamber for creating macroscale vascularatures. Reproduced with permission.^[^ ^372^ ^]^ Copyright 2017 American Chemical Society.

To establish heterogeneous tumor models with more complex structures, more and more 3D manipulation techniques have been developed.^[^ ^362^, ^363^, ^364^, ^365^, ^366^, ^367^, ^368^, ^369^, ^370^ ^]^ For example, Ayan et al. utilized an aspiration‐assisted bioprinting approach to pick and place cell spheroids into stacked structures (**Figure** **25**a).^[^ ^371^ ^]^ This versatile technique enabled the building of complex architectures used spheroid blocks. In a more interesting work, Agarwal et al. used tumor spheroids as building blocks to fill a microchamber, and subsequently introduced endothelial and stromal cells, finally obtaining a tumor model with vascular networks (Figure 25b).^[^ ^372^ ^]^ The authors used this straightforward way to engineer tumor with circulation function, which can be employed for investigating microenvironment's effect on tumor progression, invasion, and metastasis. All in all, cell spheroids or organoids, as the tissue building blocks, have been demonstrated in reconstructing the in‐vivo native microenvironment of cell‐cell interactions and ECM development.^[^ ^373^ ^]^

**Figure 26:**
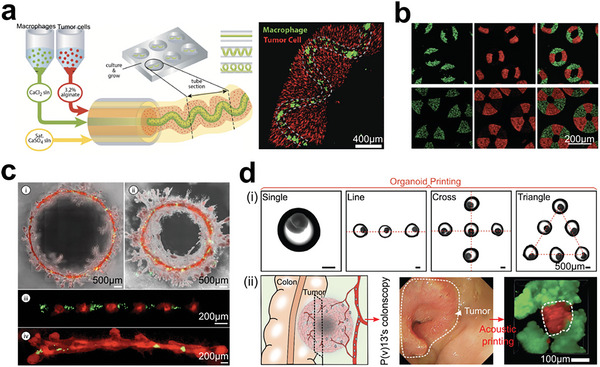
3D Tumor models with tissue‐level complex structures. a) Microfluidic printing of helical microfibers used for studying the crosstalking of tumor cells (core of microfibers) and macrophages (shell of microfibers). Reproduced with permission.^[^ ^381^ ^]^ Copyright 2015 Wiley‐VCH Verlag GmbH & Co. KGaA, Weinheim. b) Capillary microfluidic printing of microfibers with multicompartmental structures. Reproduced with permission.^[^ ^382^ ^]^ Copyright 2014 Wiley‐VCH Verlag GmbH & Co. KGaA, Weinheim. c) Bioprinting organoids into circular shapes (i, ii). MDA‐MB‐468 cells (green) (iii) and MCF‐12A cells (red) were spaced in order. Incorporation of cancer cells into organoids (iv). Reproduced with permission.^[^ ^383^ ^]^ Copyright 2019 the authors. d) Acoustic bioprinting of patient‐derived colon normal and tumor organoids to mimic colon tumor microenvironment. Reproduced with permission.^[^ ^384^ ^]^ Copyright 2022 Wiley‐VCH GmbH.

Recently, the emerging advanced techniques has accelerated the development of tumor tissue engineering, enabling the creation of tissue‐level complex structures inside tumor models.^[^ ^374^, ^375^, ^376^, ^377^, ^378^, ^379^, ^380^ ^]^ One notable example is one work that used microfluidic printing to generate helical hydrogel‐based microfibers (**Figure** **26**a), wherein tumor cells and macrophages were encapsulated and their interaction process could be studied.^[^ ^381^ ^]^ Similarly, Cheng and colleagues presented a one‐step process to continuously fabricate microfibers with designated features (Figure 26b), allowing the separation of different cancer cells into different regions.^[^ ^382^ ^]^ Bioprinting approaches have also been employed to facilitate the organization of organoids and tumors (Figure 26c).^[^ ^383^ ^]^ In addition, as demonstrated by Chen et al. who used an acoustic printing approach to precisely arrange patient‐derived normal and tumor colon organoids (Figure 26d), revealing the relationship of tumor invasion between in vitro tumor models and in vivo tumor tissue.^[^ ^384^ ^]^

#### 3.3.3 Control of Tumor Model's Structures in 4D

**Figure 27:**
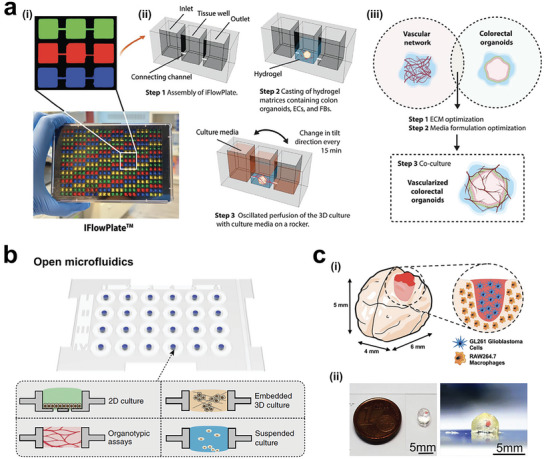
3D Tumor models with tissue‐level functional complex structures. a) 384‐well plate for culturing perfusable vascularized colon organoids. Reproduced with permission.^[^ ^396^ ^]^ Copyright 2020 Wiley‐VCH GmbH. b) Open microfluidics for establishing multilayered tissue models to study tumor‐cell‐mediated differentiation of macrophages. Reproduced with permission.^[^ ^397^ ^]^ Copyright 2019 Copyright 2020 the authors, under exclusive license to Springer Nature Limited. c) Bioprinting of glioblastoma model that contained glioblastoma cells and macrophages. Reproduced with permission.^[^ ^398^ ^]^ Copyright 2019 Wiley‐VCH Verlag GmbH & Co. KGaA, Weinheim.

Furthermore, additional components have been incorporated into tumor models in a long‐term style.^[^ ^385^, ^386^, ^387^, ^388^, ^389^, ^390^, ^391^, ^392^ ^]^ In particular, decellularized tissue parenchyma as a source of more physiologically accurate ECM upon/within which, tumour cells/organoids can be incorporated, and appropriate biochemical and biomechanical cues received.^[^ ^393^ ^]^ Recently, Edoardo et al. proposed a cancer patient‐derived ECM as the scaffolds for culturing organotypic liver tumor models. They found that decellularized ECM scaffolds can mimic the biological, biochemical, and structural characteristics of the tumor metastatic microenvironment, eventually used to assess the chemotherapy responses in a long period.^[^ ^394^ ^]^ Besides, a team developed the decellularized ECM for culturing gastrointestinal organoids which displayed superior maturation and function.^[^ ^395^ ^]^ Except the ECM, other tissue parenchyma can be incorporated into tumor models for study tumor development over time. For example, the establishment of a vascular system in colon organoids by Rajasekar et al. using a customized microfluidic chip (**Figure** **27**a).^[^ ^396^ ^]^ In a more extensive study, Yu et al. had also optimized the approach called reconfigurable open microfluidics to build up 3D tumor tissues (Figure 27b), which provided an open environment, adapting to various medium additions and cell seeding.^[^ ^397^ ^]^ Excitingly, a glioblastoma model had been established inside a bioprinted mini‐brain (Figure 27c), enabling the precise interrogation of the crosstalking between macrophages and tumor cells.^[^ ^398^ ^]^ Together, the tissue‐level engineering of tumors holds immense potential for fundamental oncology research.

## 4 Biomedical Applications of Heterogeneous Tumor Models

### 4.1 Studying Tumor‐Related Biomarkers

**Figure 28:**
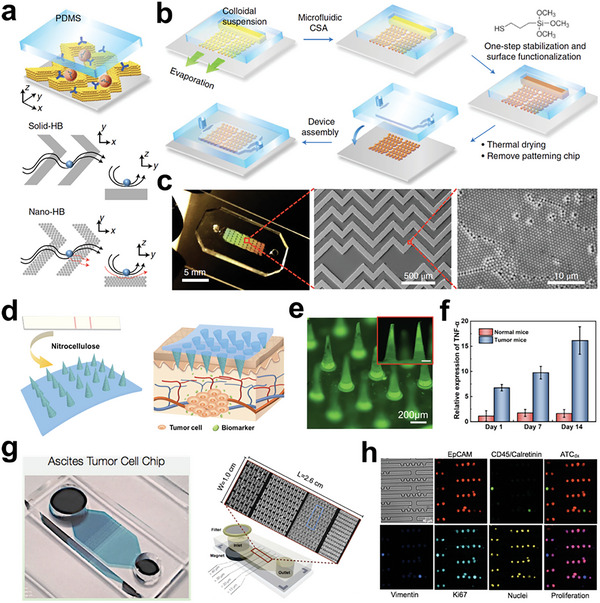
Studying tumor diseases based tumor‐related biomarkers. a) 3D nanostructured herringbone (nano‐HB) can effectively promote microscale mass transfer and enhance the binding strength of bioparticles. b) The fabrication process of 3D nano‐HB in a designed self‐assembly way. c) Nano‐HB chip manufactured by silica colloids. Reproduced with permission.^[^ ^409^ ^]^ Copyright 2019 the authors, under exclusive license to Springer Nature Limited. d) Photonic nitrocellulose‐based microneedles for detection of biomarkers in tumor interstitial fluid. e) The fluorescence image showing the capture of FITC‐labeled antibody. f) Analysis of TNF‐α expression in tumor interstitial fluid. Reproduced with permission.^[^ ^410^ ^]^ Copyright 2021 Elsevier. g) A microfluidic chip platform to enrich ascites tumor cells. The chip used a magnet to trap magnetically labeled cells. h) Microarrays fixing of targeted cells and tumor cell markers (EpCAM) can be profiled in situ. Reproduced with permission.^[^ ^411^ ^]^ Copyright 2013 National Academy of Science.

Numerous nano‐ and micro‐techniques have been developed to decode the fundamental mechanisms and track the expansion of complex artificial tumor tissues.^[^ ^399^, ^400^, ^401^, ^402^, ^403^, ^404^, ^405^, ^406^, ^407^, ^408^ ^]^ For instance, one work used a nanostructured herringbone (nano‐HB) microfluidic chip to enhance exosome capturing (**Figure** **28**a).^[^ ^409^ ^]^ They utilized a microchannel platform to induce evaporation‐driven colloidal self‐assembly (CSA) into designated nanopatterns (Figure 28b), which were modified with anti‐CD81 monoclonal antibody to capture tumor exosomes. Micropatterns with tightly‐packed colloidal particles were obtained (Figure 28c). At the microscale level, techniques have been developed to process larger samples of tumor tissues. Recently, Dong et al. developed a nitrocellulose‐based photonic bioassay for detecting biomarkers in tumor interstitial fluid (Figure 28d).^[^ ^410^ ^]^ The authors incorporated microneedle techniques to sample biomarkers from solid tumors (Figure 28e,f), enabling the quantitative detection of TNF‐α expression during tumor progression. Clinically, many point‐of‐care devices have been developed to monitor molecules during cancer treatment for patients. A microfluidic chip was designed to collect ascites tumor cells, a valuable index for reflecting tumor conditions. This miniaturized microfluidic chip with microtraps enabled on‐chip molecular profiling of ovarian cancer tissues (Figure 28g,h).^[^ ^411^ ^]^ The mentioned above impressive works bridge the gap between studying tumors and clinical therapeutics in a precise manner.

### 4.2 Quantitative Studying of Tumor Microenvironment

**Figure 29:**
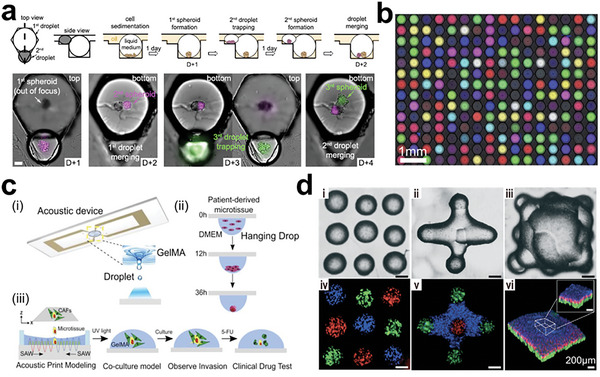
Quantitative studying of tumor microenvironment. a) Individual control and quantitation of 3D tumor spheroids using droplet microfluidics. Different spheroids can be assembled together by merging droplets. b) Multiplex drug conditions can be used to culture spheroids. Reproduced with permission.^[^ ^422^ ^]^ Copyright 2020 the authors. c) Acoustic droplet printing of patient‐derived tumor microtissues to mimic the interaction between tumor cells and cancer associated fibroblasts (CAFs). Reproduced with permission.^[^ ^423^ ^]^ Copyright 2022 The Royal Society of Chemistry. d) Acoustic droplet printing of tumor microenvironment with designated structures including grid‐like array, cross structure, and pyramid structure. Reproduced with permission.^[^ ^424^ ^]^ Copyright 2021 The Royal Society of Chemistry.

The tumor microenvironment is crucial in drug resistance, cellular communications, and progression. Quantitative analysis of tumor microenvironment can illustrate and study mechanisms of tumor development.^[^ ^65^, ^412^, ^413^, ^414^, ^415^, ^416^, ^417^, ^418^, ^419^, ^420^, ^421^ ^]^ Tomasi et al. utilized a microfluidic droplet approach to sequentially and precisely regulate culture conditions on 3D tumor spheroids (**Figure** **29**a).^[^ ^422^ ^]^ This microfluidic chip was designed with a special structure to anchor droplets in desired positions, allowing for multiple functions such as co‐culture of cell spheroids, hydrogel encapsulation, and drug testing (Figure 29b) in a quantitative manner. Recently, Chen et al. presented an acoustic bioprinting approach to reconstruct the tumor microenvironment (Figure 29c).^[^ ^423^ ^]^ This system incorporated CAFs into tumor microtissues, enabling to quantitatively study the crosstalking of CAFs and tumor cells. Furthermore, an improvement was made to the acoustic printing approach based on cell‐loaded hydrogel droplets, which established more complex architectures of tumor tissue (Figure 29d).^[^ ^424^ ^]^ The authors achieved 3D acoustic bioprinting of a spheroid‐stroma co‐culture model. The quantitative analysis of tumor models provides valuable information on tumor development.

### 4.3 Studying of Tumor Circulation, Invasion, and Metastasis In Vitro

**Figure 30:**
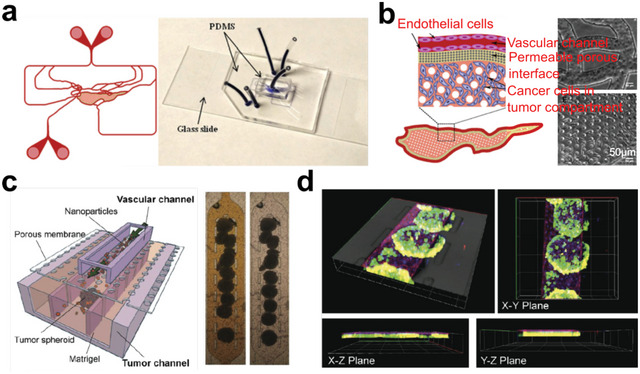
Studying of tumor circulation in in‐vitro tumor models. a) A microfluidic chip comprised of tumor and endothelial cells, forming the tumor vascular circulation system. b) Mimicking EPR effect by corporating vascular channel and tumor component. Reproduced with permission.^[^ ^434^ ^]^ Copyright 2017 the authors. c) A Tumor‐Vasculature‐on‐Chip (TVOC) for recapitulating tumor biological barrier functions. d) Representative images showing TVOC's 3D architecture with vasculature (in the top) and tumor spheroids (at the bottom). VE‐cadherin (red color); Nucleus (blue color); Tumor spheroids (green color). Reproduced with permission.^[^ ^435^ ^]^ Copyright 2018 American Chemical Society.

The study of tumor progression, including circulation, invasion, and metastasis, holds great promise for elucidating drug accumulation, interactions of tumor cells with the ECM, and normal tissues in vitro.^[^ ^425^, ^426^, ^427^, ^428^, ^429^, ^430^, ^431^, ^432^, ^433^ ^]^ Tang et al. devised a biomimetic tumor microenvironment by integrating a vascular network‐like microfluidic channel and a tumor compartment (**Figure** **30**a,b).^[^ ^434^ ^]^ This platform reconstructed enhanced permeability and retention (EPR) effect in vitro. To evaluate nanoparticles as anticancer agents, Wang and colleagues developed a Tumor‐Vasculature‐on‐Chip (TVOC), which comprises tumor spheroids and a vascular channel (Figure 30c,d).^[^ ^435^ ^]^ Nanoparticles introduced into the vascular channel accumulated in densely packed tumor spheroids, offering an opportunity to evaluate nanoparticle circulation within a solid tumor.

**Figure 31:**
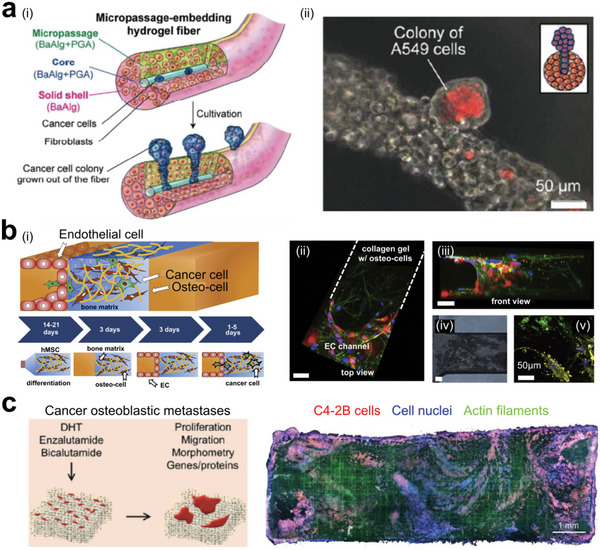
Studying of tumor invasion and metastasis in in‐vitro tumor models. a) Micropassage‐embedding hydrogel fiber loaded with cancer cells and fibroblasts. Cancer cell (A549 cells) colony was able to grow out of the fiber to mimic tumor invasion behavior. Reproduced with permission.^[^ ^441^ ^]^ Copyright 2018 The Royal Society of Chemistry. b) Microfluidic chip for modeling breast cancer metastasis to bone. Reproduced with permission.^[^ ^443^ ^]^ Copyright 2013 Elsevier Ltd. c) Modeling of prostate cancer osteoblastic metastases. Reproduced with permission.^[^ ^444^ ^]^ Copyright 2021 the authors, under the exclusive license to American Association for the Advancement of Science.

Tumor invasion and metastasis have also been studied in engineered tumor models.^[^ ^436^, ^437^, ^438^, ^439^, ^440^ ^]^ Sugimoto et al. developed a novel platform for evaluating cancer cell invasion in a 3D tissue‐like environment (**Figure** **31**a).^[^ ^441^ ^]^ The authors created a composite microfiber containing tumor cells in the core region and fibroblasts in the shell region, with a micropassage within the fiber enabling tumor cells to grow out, thus mimicking cancer invasion through the extracellular matrix. Besides, tumor invasion models have great potential in the precise drug evaluation, due to therapeutic drug concentrations not reaching the infiltrative tumor. For example, Puls et al. developed a novel 3D tumor‐tissue invasion model for high‐throughput, high‐content drug screening.^[^ ^442^ ^]^ By using next‐generation invasion models that better replicate the complexity of the tumor microenvironment, researchers can gain a deeper understanding of tumor invasion patterns and develop more effective therapeutic strategies. These models can aid in the design of clinical trials that consider drug penetration issues and potentially improve the success of chemotherapy and other treatments for invasive tumors.

Furthermore, microfluidics can provide a powerful system for studying tumor metastasis through parenchyma. Bersini et al. demonstrated that a microfluidic chip can be used to replicate cancer cell metastasis into bone tissue (Figure 31b).^[^ ^443^ ^]^ Another work presented an in‐vitro model that included mineralized metastatic organ, corporating osteoprogenitor cells with tumor cells. This model can assess the effects of drugs such as antiandrogens, bicalutamide, and enzalutamide (Figure 31c).^[^ ^444^ ^]^ To precisely control over factors such as flow, shear stress, and chemical gradients, which can influence tumor migration and invasion, researchers are improving the complexity and functionality of tissue chips to better mimic in vivo parenchymal conditions.^[^ ^445^ ^]^ Collectively, the profiling of tumor progression can contribute to a better understanding of cancer biology.

### 4.4 Studying of Tumor Mechanics

**Figure 32:**
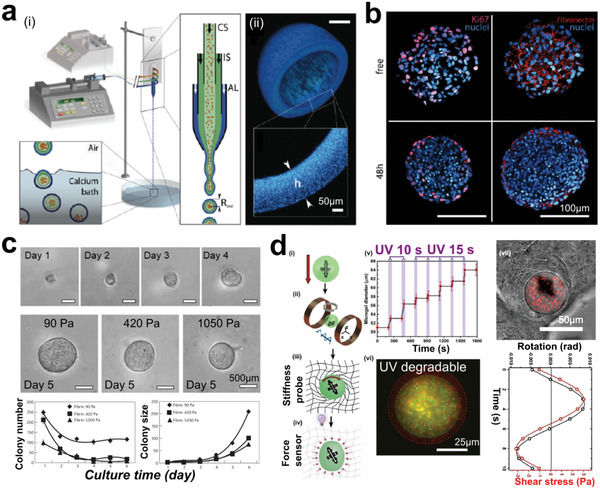
Studying of tumor mechanics. a) Tumor spheroids encapsulated in microfluidics‐generated droplets that showed different mechanical cofinements to spheroids. b) Representative images showing the cellular organization in free (top) and confined (bottom) spheroids. DAPI (blue); KI67 (magenta); Fibronectin (red). Reproduced with permission.^[^ ^466^ ^]^ Copyright 2013 National Academy of Science. c) Multicellular tumor spheroids formation and growth in 3D soft fibrin gels of different stiffness. Reproduced with permission.^[^ ^467^ ^]^ Copyright 2012 Macmillan Publishers Limited. d) Magnetic microrobot quantifying stiffness and forces of tumor colonies. i–iv) Quantification processes including stiffness measurement by applying torque and traction force measurement by analyzing the deformation of microgel after the softening step. v–vi) The property changes of microgels after UV irradiation. vii) Representative image showing a microrobot probe inside a 3D tumor‐repopulating cells (top) and the measurement of probe angular rotation in response to a sinusoidal magnetic twisting (bottom). Reproduced with permission.^[^ ^468^ ^]^ Copyright 2023 the authors, under the exclusive license to American Association for the Advancement of Science.

The mechanics of tumor tissues are essential in tumor progression and cancer therapeutic responses.^[^ ^446^, ^447^, ^448^, ^449^, ^450^, ^451^, ^452^, ^453^, ^454^, ^455^, ^456^, ^457^, ^458^, ^459^ ^]^ The tumor microenvironment is highly heterogeneous, and several factors contribute to its mechanical properties, including interstitial pressure and matrix conditions.^[^ ^446^, ^460^, ^461^, ^462^, ^463^, ^464^, ^465^ ^]^ To well understand the relationship between tumor growth and ECM stiffness, Alessandri and colleagues presented a microfluidic method to generate cell‐loaded droplets with controllable hydrogel shells (**Figure** **32**a).^[^ ^466^ ^]^ By analyzing the mechanics of encapsulated spheroids, the authors found that cell division and fibronectin primarily occur in the peripheral rim of spheroids in the hydrogel‐confined condition (Figure 32b). To mimic the in vivo ECM condition, Liu et al. presented an in vitro system to culture tumorigenic cells in fibrin matrices (Figure 32c).^[^ ^467^ ^]^ The authors demonstrated that soft fibrin gels can enhance tumor cell's growth. To precisely measure the mechanics of tumor tissue, Mohagheghian et al. used a magnetic microrobot probe to quantitatively assess the stiffness and surrounding traction forces of tumor tissue (Figure 32d).^[^ ^468^ ^]^ This microrobot probe with controllable functionality via magnetic fields could be useful for studying the mechanoregulation of the tumor microenvironment. Overall, the assessment of mechanical forces in tumor tissue could provide insights into tumor progression and further improve cancer therapeutics.

### 4.5 Studying of Immunology

**Figure 33:**
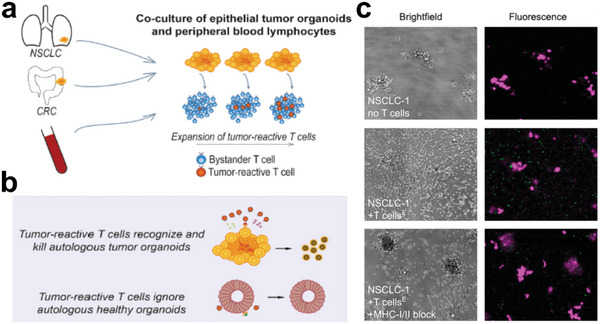
Studying of immunology in tumor models by activating immune‐tumor signaling. a) Peripheral blood lymphocytes and epithelial tumor organoids were cultured together, enabling the expansion of tumor‐reactive T cells. b) Tumor‐reactive T cells’ cytotoxicity had selectivity to tumor organoids rather than healthy organoids. c) The typical images showing autologous tumor‐reactive T cells had the cytotoxicity to tumor organoids after 72 h. Organoids were stained with CellTrace Yellow (magenta); Apoptotic cells (green). Reproduced with permission.^[^ ^480^ ^]^ Copyright 2018 Elsevier Inc.

The activation and engineering of immune cells to target tumor cells is a complex and heterogeneous process that requires quantitative profiling of the immune cell populations in the tumor microenvironment.^[^ ^469^, ^470^, ^471^, ^472^, ^473^, ^474^, ^475^, ^476^, ^477^, ^478^, ^479^ ^]^ Tissue‐engineered models can incorporate immune and stromal cell populations to study these interactions and their effects on tumor growth and evolution. Recently, tumor organoids have been used as a platform to activate peripheral blood lymphocytes (PBLs) and assess their anti‐cancer abilities (**Figure** **33**a).^[^ ^480^ ^]^ The activated T cells ignored autologous healthy organoids, demonstrating antigen‐specific CD8^+^ T cell response (Figure 33b). Furthermore, PBLs were cultured with autologous tumor organoids originated from human non‐small‐cell lung cancer (NSCLC) tissues. These PBLs showed a tumor‐reactive capacity, reducing the viability of tumor organoids (Figure 33c).

**Figure 34:**
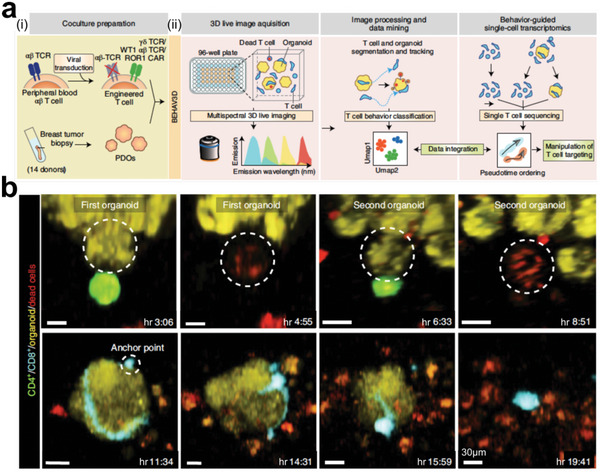
Evaluating of immunocyte's cytotoxicity in tumor models. a) A platform for investigating the T cells’ behaviors inside patient‐derived tumor organoids. b) Representative images showing a CD4^+^ metabolome‐sensing engineered T cell (TEG) killed two tumor cells in two different tumor organoids. A CD8^+^ TEG completely killed a tumor organoid after 11 h (bottom). Reproduced with permission.^[^ ^483^ ^]^ Copyright 2022 the authors.

Another cancer targeting therapeutics is chimeric antigen receptor (CAR) T‐cell treatment that sometimes shows low efficacy and serious side effects. Therefore, profiling the dynamics and function of CAR‐T cells is essential for optimizing this therapy.^[^ ^132^, ^481^, ^482^ ^]^ Dekkers et al. developed a system to investigate the dynamic crosstalking of patient tumor organoids and immune cells through real‐time visualization and transcriptomics (**Figure** **34**).^[^ ^483^ ^]^ When cultured with patient‐derived cancer organoids, the authors found that, more than 150000 engineered T cells showed the specific performances. The authors demonstrated a typical cluster containing T lymphocytes with continuous cytotoxicity, called a “super engager” behavioral. Additionally, tumor metabolome‐sensing engineered T lymphocytes (TEGs) are another concept being studied. Together, tumor organoids provide a valuable in vivo‐like tissue microenvironment for the assessment of immune cell functions.

### 4.6 Multi‐organ Chip Containing Tumor Tissues

**Figure 35:**
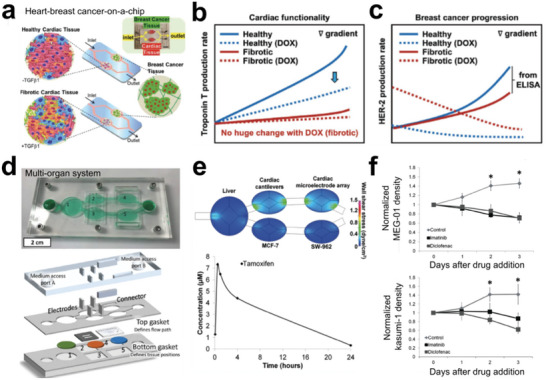
Multi‐organ chip with tumor tissues. a) Heart‐breast cancer model for assessing triggered cardiotoxicity under cancer chemotherapy. b) Troponin T rate in normal and diseased cardiac tissues on dual chips with and without the DOX treatment. c) Predicted HER‐2 rate in healthy and fibrotic cardiac tissues on dual chips with and without the treatment of DOX. Reproduced with permission.^[^ ^490^ ^]^ Copyright 2020 Wiley‐VCH GmbH. d) Multi‐organ system for evaluating efficacy of anti‐cancer therapy. Chamber 1 (hepatocytes on coverslips); Chamber 2 and 4 (cardiac cantilevers and microelectrode arrays (MEAs)); Chamber 3 and 5 housed SW‐962 and MCF‐7 cancer cells, respectively. e) Computational fluid dynamics (CFD) modeling for determining pharmacodynamic (PD) parameters (top). Sample aliquots aquired from the chip were analyzed by HPLC to obtain an AUC tamoxifen concentration curve (bottom). f) Effect of imatinib and diclofenac on growth capability of two bone marrow cell lines (megakaryocyte, Kasumi‐1 cells). Reproduced with permission.^[^ ^491^ ^]^ Copyright 2019 the authors, under the exclusive license to American Association for the Advancement of Science.

Tumors have a complex relationship with other organs, often invading and spreading to other parts of the body.^[^ ^484^, ^485^, ^486^, ^487^, ^488^, ^489^ ^]^ In addition, tumors can produce hormones and chemicals that impact the functioning of other organs. In the clinic, chemotherapy‐induced cardiotoxicity (CIC) is a significant concern. To address this issue, one work presented a heart‐breast cancer model on a chip for permit the metronomic drug soding regimes (**Figure** **35**a), that contained patient‐derived breast cancer cells and cardiac tissues.^[^ ^490^ ^]^ By studying the Troponin T's secretion degree, typically representing the cardiac functionality, the authors found that pre‐existing cardiac fibrosis resulted in lower Troponin T levels than that in healthy cardiac components, no matter doxorubicin (DOX) immersed. Additionally, DOX‐treated normal cardiac tissues exhibited a decline in producing Troponin. However, fibrotic cardiac tissues showed very small difference in the Troponin T production (Figure 35b). Further, these two kinds of cardiac tissues without DOX showed a positive relationship in HER‐2 secretary efficacy. However, both conditions treated with DOX displayed a negative gradient of HER‐2 production rate. Importantly, normal cardiac tissues displayed a greater HER‐2 secretary rate gradient regardless of treating DOX than the diseased cardiac tissues (Figure 35c). Multi‐organ systems consisting of functional organ modules have also been developed, such as the microfluidic device created by McAleer et al. that reconstructed bone marrow/liver and cancer/cardiomyocyte/hepatocyte systems (Figure 35d).^[^ ^491^ ^]^ This system allowed for analysis of drug concentration (Figure 35e) and demonstrated the cytostatic effects of bone marrow‐derived cells on cancer cells observed clinically (Figure 35f). These in vitro tumor models provide valuable insights into the relationship between tumors and other organs and can aid in developing useful cancer therapeutics.

### 4.7 Drug Screening

**Figure 36:**
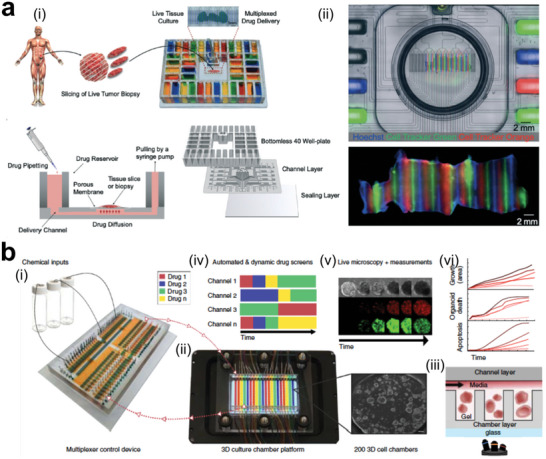
Drug screening with a high‐throughput performance on tumor models. a) A microfluidic system for testing multiple anti‐cancer drugs on tumor slices (i), and fluorescent images showing delivery of Hoechst (blue), Cell Tracker Green (green), and Cell Tracker Orange (red) to glioblastoma multiforme slices. Reproduced with permission.^[^ ^273^ ^]^ Copyright 2019 The Royal Society of Chemistry. b) Automated microfluidic system for multiplex drug screening of tumor organoids. i) The programmable membrane‐valve‐based microfluidic chip provides separated and automated stimulation to culture chambers. ii,iii) Separated 3D culture chambers and the cross‐section of the two‐layer chamber device. iv) 30 chemical inputs and 30 outlets of the automatic programmed control device. v,vi) 3D cultures like organoids can be continuously observed under the dynamic drug treatments. Reproduced with permission.^[^ ^502^ ^]^ Copyright 2020 the authors.

Tumor models have been widely employed in drug screening according to their capacity of providing controlled experimental conditions and data on the effects of drugs on cancer cells’ growth and behavior.^[^ ^234^, ^263^, ^492^, ^493^, ^494^, ^495^, ^496^, ^497^, ^498^, ^499^, ^500^, ^501^ ^]^ They can also be used to evaluate cancer development and assess the efficiency of potential treatments. Researchers can study how tumors evolve resistance mechanisms in response to treatment and explore strategies to overcome resistance. Rodriguez et al. developed a microfluidic system including multiple microchannels to load various drugs, enabling real‐time measurements of drug function on tumor slices (**Figure** **36**a).^[^ ^273^ ^]^ The authors also presented useful guidelines for drug reactions about cell death and proliferation, containing cell viability analysis conducted directly on chip. To enhance drug screening efficacy using tumor models, an automatic microfluidic platform was developed with high throughput and precision performance (Figure 36b).^[^ ^502^ ^]^ This system was built with automated fluidic architecture, facilitating the controllable changes toward the medium environment and enabling the dynamic screening of various drug combinations. These works aimed to improve the quality of drug screening by maintaining tissue microenvironment, reconstructing the condition of drug resistance, and corporating more intelligence systems.

**Figure 37:**
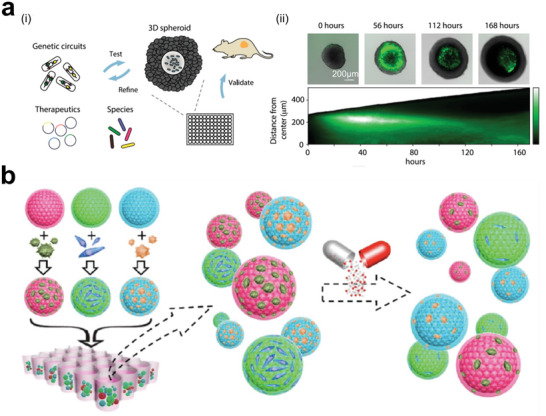
Drug screening on tumor models in physiologically relevant environments. a) A 3D multicellular model for rapidly screening microbial therapies. Reproduced with permission.^[^ ^509^ ^]^ Copyright 2019 National Academy of Science. b) Cell cultured on photonic crystal barcodes for screening anti‐cancer drugs. Reproduced with permission.^[^ ^510^ ^]^ Copyright 2016 American Chemical Society.

Despite advances in drug screening by using in vitro tumor models, the acute high‐dose cytotoxic assessment is still limited due to the viability period of these models. To create more physiologically relevant environments for studying long‐term drug effects, more and more approaches have emerged.^[^ ^503^, ^504^, ^505^, ^506^, ^507^, ^508^ ^]^ Recently, one work developed a bacteria‐in‐spheroid coculture (BSCC) platform for rapid screening of bacterial anti‐cancer therapies (**Figure** **37**a).^[^ ^509^ ^]^ By confining bacteria in spheroids, the authors successfully achieved diverse cells cultured with bacteria. Based on this platform, the authors tested the anticancer efficacy of gene‐editted clinically relevant *S. typhimurium*. Besides, via on‐chip culture of tumor organoids, real‐time and quantitative analysis of drug efficacy can be achieved. Fu et al. employed photonic crystal barcodes to label different cell types, distinguishing their heterogeneous responses to drug treatments (Figure 37b).^[^ ^510^ ^]^ These barcodes provided an in vivo‐like microenvironment for cell adhesion and proliferation. This cell spheroids‐on‐barcodes platform exhibited the capacity to provide ECM condition and monitoring drug treatments.

**Figure 38:**
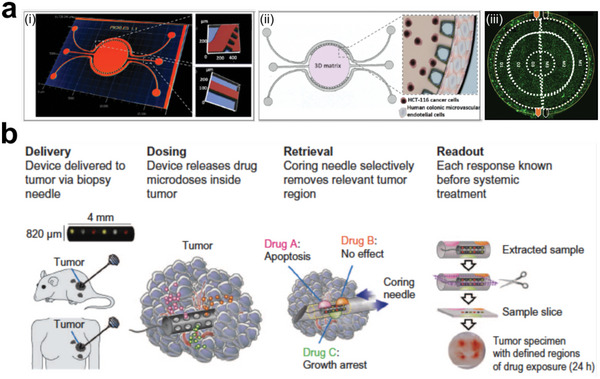
Drug screening using tumor models. a) A 3D colorectal tumor chip enabling the precise oncology nanomedicine. Reproduced with permission.^[^ ^526^ ^]^ Copyright 2019 the authors, under the exclusive license to American Association for the Advancement of Science. b) An implantable and recyclable microdevice for testing in vivo drug sensitivity of tumor tissue. Reproduced with permission.^[^ ^527^ ^]^ Copyright 2015 the authors, under the exclusive license to American Association for the Advancement of Science.

So far, nanomedicine has been widely applied in precise tumor therapeutics and shows great potential in the targeted killing of tumor cells.^[^ ^511^, ^512^, ^513^, ^514^, ^515^, ^516^, ^517^, ^518^, ^519^, ^520^, ^521^, ^522^, ^523^, ^524^, ^525^ ^]^ To evaluate the efficacy of nanoscale drugs, various tumor models have been used and even several in‐vivo techniques have been developed. Recently, one study developed a colorectal tumor‐on‐a‐chip system for precisely assessing delivery processes of drug‐loaded nanoparticles (**Figure** **38**a).^[^ ^526^ ^]^ This system can mimic the in vivo‐like drug penetration process and recapitulate cell migration processes. The authors demonstrated that their platform had the potential to study the EPR effect in the vascular network. However, most drug screening approaches are performed in vitro, and predicting drug efficacy in clinical trials occasionally fails. Jonas et al. developed an implantable microscale device that delivers precise doses of drugs into a tumor and simultaneously assesses drug efficacy in vivo (Figure 38b).^[^ ^527^ ^]^ Together, these approaches to assessing anti‐cancer drug efficacy facilitate clinical diagnosis and treatment, and drug development.

### 4.8 Tumor Models Meet Artificial Intelligence

**Figure 39:**
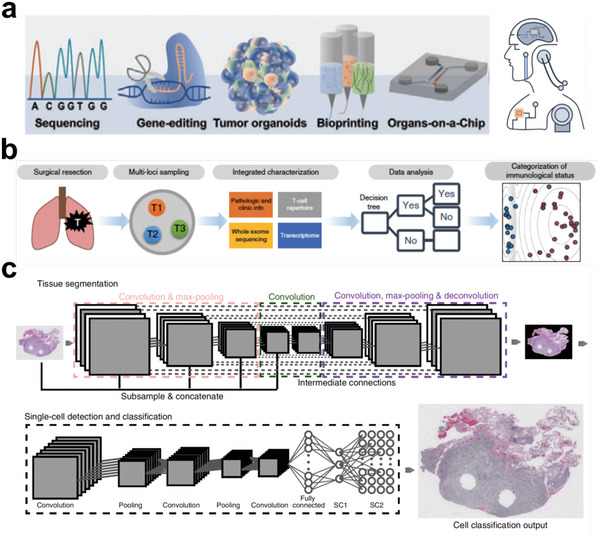
Tumor models meet artificial intelligence. a) Artificial intelligence used in molecular, cellular, and technically structural studying of oncology. Reproduced with permission.^[^ ^528^ ^]^ Copyright 2020 the authors. b) A machine learning algorithm developed to assess immunophenotypes. Reproduced with permission.^[^ ^531^ ^]^ Copyright 2018 the authors. c) A computational pathology deep‐learning pipeline for dissecting heterogeneous non‐small cell lung cancers (NSCLCs). Hematoxylin and Eosin (H&E) and triplex CD4/CD8/FOXP3 IHC slides were produced from diagnostic blocks that represented standard clinical sampling. Reproduced with permission.^[^ ^535^ ^]^ Copyright 2020 the authors, under exclusive license to Springer Nature Limited.

Artificial intelligence (AI) has been developed as the useful tool to analyze and interpreting data from tumor models, allowing for a deeper understanding of cancer and its potential treatments (**Figure** **39**a).^[^ ^528^ ^]^ AI algorithms are being utilized to identify predictive markers and extract novel insights from the data, such as elucidating the immunological signatures and assisting in determining the most effective treatment strategy for a patient.^[^ ^529^, ^530^ ^]^ Moreover, AI models can help researchers predict how tumors may evolve over time and evaluate the success of various treatments in clinical trials. For instance, a machine learning algorithm was employed to classify the immunological status based on features from transcriptome profiling, T cell repertoire analysis, and whole exome sequencing of patient tumor tissues (Figure 39b).^[^ ^531^ ^]^ This study demonstrated immune microenvironment displayed huge differences in spatial level and that entire mutations correlated with T lymphocyte's expansion. Overall, the use of AI in tumor modeling can be significantly advance our knowledges of cancer and ultimately improve patient outcomes.^[^ ^532^, ^533^, ^534^ ^]^

Recently, plenty of AI‐based advancements have been made to investigate the role of immune cell spatial heterogeneity within tumor tissues. However, a comprehensive understanding in immune escape through cancer spatial structuers requires the investigation of other tumor cells. Recently, AbdulJabbar and colleagues presented a novel approach to spatially map tumor cells, T cells, stromal cells, macrophages in H&E‐stained images (Figure 39c).^[^ ^535^ ^]^ The proposed intelligence approach comprises several components: i) segmentation of tissue regions, which utilizes multiresolution image features to decrease noises or artefacts, enabling better cell identification and precise categorization in deep neural network; ii) the cell identification system, which can predict the possibility that each pixel relates to the nucleus center in tissue areas; and iii) a cell categorization framework employing a neighboring ensemble predictor classifier, in conjunction with SCCNN, for classifying different cell types. The results of this approach highlighted the clinical importance of immune cold areas, which can show immune‐evading subclones, and thus warrant deeper studying into the fundamentals that can lead to the spatial heterogeneity of immunocytes. Taken together, artificial intelligence has the potential to significantly support tumor engineering and therapeutics with high efficacy.

## 5 Challenges and Future Directions

Tumor models have shown great potential in fundamental research, disease research, and drug discovery, as we mentioned above. The inconsistent tumor engineering process leads to variations in tumor configurations, like cell‐cell contacts, cell‐matrix communications, and tissue architecture. Tumor engineering strategies encompass a wide range of approaches, each with its own strengths and limitations. For example, one emerging tumor model, organoid, is a 3D structure grown from patient‐derived cells and can replicate the complexity of the original tumor. They allow for high‐throughput drug screening and personalized medicine approaches. However, organoids may lack immune system components and the full tumor microenvironment. They are also limited by the availability of patient samples. The choice of strategy depends on the specific research goals and the aspects of tumor biology being studied.

To ensure faithful and repeatable results, tumor models must be calibrated to the same state of tumor development. However, the lack of standardization in the engineering processes has hindered the development of these models, and their precise utilization requires calibration of the dynamics of the tumor microenvironment over time.​ For instance, ensuring that tumor spheroids are of the same size can improve the accuracy of anti‐cancer drug therapy. Controlling the growth rate of tumor spheroids is also necessary in drug‐testing tumor models. Furthermore, different stages of tumor progression entail various biological events, which can vary the detecting index of tumor phenotypes. So, it is essential to address the impact of the dynamics of tumor development on the final results of tumor investigations. Collectively, the development of uniform tumor models is critical for future research.

The engineering strategy and applications of tumor models should be linked with a more efficient intelligent system for precisely and comprehensively evaluating tumor biology. First, most current tumor models lack the precise and quantitative characterization technique, resulting in the insufficient acquirement and delayed analysis of tumor biological events, such as the dynamic secretion of signal molecules, functions of extracellular vesicles, and cell proliferation. Except for the need to advance characterization techniques, more engineering approaches are supposed to be developed to improve the reconstruction of the tumor model's structure. For example, precise control of growth factors’ distributions, expression of cell organization‐related proteins, and establishment of functional vascular constructs is required. Excitingly, there are two promising engineering approaches in promoting the development of tumor model engineering. One is a tumor organoid, derived from the primary tumor tissues, that possesses the intra‐ and intertumoral heterogeneity of the primary tumor microenvironment. 3D tumor organoids exhibit in vivo‐like cell differentiation and cell proliferation, enabling the investigation of tumors from the gene, submolecule, and cell to tissue level. Moreover, To prepare for the forthcoming era of artificial intelligence, AI‐based algorithms, the second one, can be utilized for standardizing the fabrication process and controlling the final dimension and components of tumor models.^[^ ^536^ ^]^ Additionally, with guidance from machine learning, the applications of engineered tumor models can be significantly improved to yield more reliable data. By leveraging existing and exploring more advanced technologies, researchers can reconstruct more physiological functions by integrating multiple organs or tissues. Thus, the function of tumor models with heterogeneous microenvironment can be strongly enriched in the future research, and ultimately discovering novel diagnostic markers and improving the therapeutic efficacy.

## Conflict of Interest

The authors declare no conflict of interest.
